# System-wide identification of novel de-ubiquitination targets for USP10 in gastric cancer metastasis through multi-omics screening

**DOI:** 10.1186/s12885-024-12549-3

**Published:** 2024-06-27

**Authors:** Zhi Zeng, Yina Li, Heng Zhou, Mingyang Li, Juan Ye, Dan Li, Yuxi Zhu, Yonggang Zhang, Xu Zhang, Yunchao Deng, Juan Li, Lijuan Gu, Jie Wu

**Affiliations:** 1 Department of Pathology, Renmin Hospital of Wuhan University, Wuhan, Hubei China; 2 Department of Anesthesiology, Renmin Hospital of Wuhan University, Wuhan, Hubei China; 3 Department of Neurosurgery, Renmin Hospital of Wuhan University, Wuhan, Hubei China; 4 Department of Pharmacy, Huazhong University of Science and Technology Hospital, Wuhan, Hubei China; 5 Department of Pharmacy, Renmin Hospital of Wuhan University, Wuhan, Hubei China; 6 Division of Biostatistics, College of Public Health, The Ohio State University, Columbus, OH USA; 7 Department of Gastroenterology, Renmin Hospital of Wuhan University, Wuhan, Hubei China; 8 Hubei Key Laboratory of Resources and Chemistry of Chinese Medicine, School of Pharmacy, Hubei University of Chinese Medicine, Wuhan, Hubei China; 9 Central Laboratory, Renmin Hospital of Wuhan University, Wuhan, Hubei China

**Keywords:** USP10, Gastric cancer, TNFRSF10B, EMT, Migration and invasion, Ubiquitination

## Abstract

**Objective:**

Ubiquitin-specific peptidase 10 (USP10), a typical de-ubiquitinase, has been found to play a double-edged role in human cancers. Previously, we reported that the expression of USP10 was negatively correlated with the depth of gastric wall invasion, lymph node metastasis, and prognosis in gastric cancer (GC) patients. However, it remains unclear whether USP10 can regulate the metastasis of GC cells through its de-ubiquitination function.

**Methods:**

In this study, proteome, ubiquitinome, and transcriptome analyses were conducted to comprehensively identify novel de-ubiquitination targets for USP10 in GC cells. Subsequently, a series of validation experiments, including in vitro cell culture studies, in vivo metastatic tumor models, and clinical sample analyses, were performed to elucidate the regulatory mechanism of USP10 and its de-ubiquitination targets in GC metastasis.

**Results:**

After overexpression of USP10 in GC cells, 146 proteins, 489 ubiquitin sites, and 61 mRNAs exhibited differential expression. By integrating the results of multi-omics, we ultimately screened 9 potential substrates of USP10, including TNFRSF10B, SLC2A3, CD44, CSTF2, RPS27, TPD52, GPS1, RNF185, and MED16. Among them, TNFRSF10B was further verified as a direct de-ubiquitination target for USP10 by Co-IP and protein stabilization assays. The dysregulation of USP10 or TNFRSF10B affected the migration and invasion of GC cells in vitro and in vivo models. Molecular mechanism studies showed that USP10 inhibited the epithelial-mesenchymal transition (EMT) process by increasing the stability of TNFRSF10B protein, thereby regulating the migration and invasion of GC cells. Finally, the retrospective clinical sample studies demonstrated that the downregulation of TNFRSF10B expression was associated with poor survival among 4 of 7 GC cohorts, and the expression of TNFRSF10B protein was significantly negatively correlated with the incidence of distant metastasis, diffuse type, and poorly cohesive carcinoma.

**Conclusions:**

Our study established a high-throughput strategy for screening de-ubiquitination targets for USP10 and further confirmed that inhibiting the ubiquitination of TNFRSF10B might be a promising therapeutic strategy for GC metastasis.

**Supplementary Information:**

The online version contains supplementary material available at 10.1186/s12885-024-12549-3.

## Introduction

Ubiquitin-specific protease 10 (USP10) is a typical de-ubiquitinase that cleaves ubiquitin from ubiquitin-conjugated protein substrates to enhance protein stability [1–3]. Recent studies have shown that USP10 plays a double-edged sword role in human tumors [4]. For example, USP10 directly interacts with PTEN through de-ubiquitination to stabilize PTEN protein, thereby inhibiting the invasion of lung cancer cells [3]. Conversely, USP10 can also directly interact with Smad4 and stabilize it, resulting in increasing the metastatic ability of hepatocellular carcinoma [5]. In addition, Yuan et al. reported that USP10 acts as a tumor suppressor in renal cell carcinoma CAKI-1 and CAKI-2 cells with wild-type p53, but has the opposite effect in renal cell carcinoma 786O cells with mutant type p53 [6]. Therefore, USP10 cannot be simply defined as a tumor suppressor gene or an oncogene, and its role may be directly related to the function of its deubiquitinated protein, that is, USP10 regulates the biological behavior of tumor cells in a context-dependent manner [7]. If the gene deubiquitinated by USP10 is an oncogene, it may lead to tumor progression, and if the gene deubiquitinated by USP10 is a tumor suppressor gene, it may lead to tumor suppression [7].

Gastric cancer (GC) is the most common malignant gastrointestinal cancer worldwide [8]. Despite the therapeutic strategies being improved for GC, the prognosis of advanced GC patients is still poor [9]. Exploring novel sensitive markers and therapeutic targets for the diagnosis, treatment, and prevention of GC is essential. Recently, Li et al. reported that CircCOL1A2 downregulates the ubiquitination level of RFC2 by upregulating the expression of USP10, promoting the invasion and migration of GC cells [10]. Zhang et al. found that LINC00240 promotes GC progression through USP10-mediated DDX21 stabilization [11]. However, our previous studies showed that USP10 expression is downregulated in a large number of GC clinical samples and cell lines, and GC patients with low USP10 expression exhibit a marked propensity toward gastric wall invasion, lymph node metastasis, highly malignant biological behavior, and poor survival [12]. Meanwhile, Cheng et al. reported that the DZNep reduces the binding of ubiquitin to p53 by upregulating USP10, thereby inhibiting the degradation of wild-type p53 protein and promoting GC cell death [13]. Thus, we speculate that USP10 may also play a double-edged sword role in the occurrence and development of GC. The key to understanding the seemingly contradictory research results mentioned above is to elucidate which proteins can be deubiquitinated by USP10 at different stages of GC. In addition to RFC2, DDX21, and p53, are there any other key deubiquitinating substrates of USP10 involved in the progression of GC? Can we use high-throughput methods to quickly screen these deubiquitinating substrates? Among them, which ones may function as tumor suppressors and further become potential targets for the treatment of GC?

Epithelial-mesenchymal transition (EMT) is a crucial program for cancer cell invasion and metastasis [14, 15]. The down-regulation of the epithelial cell adhesion molecules, including E-cadherin and tight junction proteins, and up-regulation of the mesenchymal cell markers N-Cadherin and Vimentin are the major hallmarks of EMT [16, 17]. Among them, E-cadherin is a member of the Type I classical cadherin family, which forms the structure for establishing and maintaining cell-cell interactions [18]. Accumulating evidences suggest that E-cadherin plays an important role in the EMT process of GC [18, 19]. Moreover, our previous studies found that the expression of USP10 was positively correlated with the expression of E-cadherin [12]. These results indicate that USP10 may participate in the invasion and metastasis process of GC by regulating EMT related proteins.

The integration of the proteome with the ubiquitinome enables high-throughput identification of substrates for the ubiquitin-specific proteases, which provides a powerful role in uncovering their biological function involved in pathological processes [20, 21]. Therefore, in the present study, we utilized a series of proteome, ubiquitinome, and transcriptome to system-wide identify novel de-ubiquitination targets for USP10 in GC cells. Subsequently, we focused on investigating the impact of USP10 and its de-ubiquitination targets on EMT process to uncover their regulatory mechanisms in GC metastasis.

## Methods and materials

### Cell culture and cell transfection

Human gastric cancer cell lines (AGS or MKN45) were purchased from the Cell Bank of the Shanghai Institute for Biological Science (Shanghai, China), and Homo sapiens embryonic kidney cell line 293T was purchased from the China Center for Type Culture Collection (Wuhan, Hubei, China), which were used in the experiments and cultured under standard conditions. 5 × 10^5^ AGS or MKN45 cells were seeded into a 6-well plate per well for 24 h prior to transfection and the cells were cultured overnight. When the cells had grown to 50–60% confluence, the si-NC mimics, si-USP10, or si-TNFRSF10B (GenePharma Co., Ltd., Suzhou, China) were transfected into cells with InvitroRNA™ (InvivoGene Biotechnology Co., Ltd., Suzhou, China) according to the manufacturer’s instructions. Then, knockdown efficiency was confirmed by Western blot. After 48–72 h of transfection, cells were collected for the following experiments. siRNAs were designed by and purchased from GenePharma (Suzhou, China), and the siRNA sequences were showed in Table S1. 5 × 10^5^ AGS, MKN45, or 293T cells were seeded into a 6-well plate per well for 24 h prior to transfection, and the cells were cultured overnight. When the cells had grown to 60–70% confluence, the overexpression plasmid of USP10 (GV657-USP10) or empty vector (GV657) was transfected into cells with Neofect^®^ DNA transfection reagent (Neofect (Beijing) Biotech Co., Ltd., Beijing, China) according to the manufacturer’s instructions. After 48 h of transfection, the green fluorescent could be detected under the inverted fluorescence microscope (Olympus Corporation, Japan), and overexpression efficiency was confirmed by Western blot, then cells were collected for the following experiments including the wound healing assay, transwell invasion assay, co-immunoprecipitation assay, and so on. The GV657-USP10 or GV657 were designed by and purchased from GeneChem (GeneChem Medical Technology Co., Ltd., Shanghai, China). The CDS (2397 bp) of ubiquitin carboxyl-terminal hydrolase 10 isoform 2 (NM_005153.3) was cloned and inserted into GV657 plasmid to construct GV657-USP10.

### Protein extraction and western blot (WB) analysis

A detailed description of protein extraction and WB analysis was shown in the Supplementary Materials and Methods. The anti-USP10 (ab109219, Abcam, Cambridge, UK), E-cadherin (EM0502, Huabio, Hangzhou, China), Twist1 (EM1710-24, Huabio, Hangzhou, China), DR5 (TNFRSF10B) (sc-166,624, Santa Cruz Biotechnology, Dallas, TX, USA), TRAP95 (MED16) (sc-130,439, Santa Cruz Biotechnology, Dallas, TX, USA), GAPDH (GB11002, Servicebio Technology, Wuhan, China), β-tubulin (abs830032, Absin Bioscience Inc., Shanghai, China), or Vinculin (ab129002, Abcam, Cambridge, UK) was used. The blots were cut prior to hybridization with antibodies, so original images of full-length blots cannot be provided, but the membrane edges of the images of the original blots in Supplementary Material 4 were visible.

### Co-immunoprecipitation (Co-IP) assay

After 293T cells were transfected with GV657-USP10 plasmid (Flag tag), the cells were collected for Co-IP assay using a commercial kit (abs955, Absin Bioscience Inc., Shanghai, China). The cell lysates were incubated with anti-Flag (USP10) antibody, and control IgG respectively at 4 ℃ overnight followed by the treatment with protein A/G agarose beads and further incubated at 4 ℃ overnight. Then, Lysates were centrifuged and the precipitate was washed thrice with cold wash buffer. Finally, the immunoprecipitated proteins of USP10 and TNFRSF10B were detected by Western blot analysis.

### Sample preparation for high-throughput detection

6 × 10^8^ 293T cells were seeded into a 1-well plate prior to transfection. When the cells reached to 70–80% confluence, GV492-USP10 (GV492 was the empty vector), psPAX2, and pMD2.G (mole ratio 1: 1:1) (Genechem, China) were transfected into cells with lipofectamine™ 2000 (11,668,019, invitrogen, Thermo Fisher Scientific, Carlsbad, CA, USA) according to the manufacturer’s instructions. After GV492-USP10 was successfully packaged into recombinant lentiviral particles, 5 × 10^4^ AGS cells were seeded into a 6-well plate. When the cells had grown to 20–30% confluence, AGS cells were infected with GV492 or GV492-USP10 virus. Then, the stable AGS cells were screened with 1.5 ug/mL puromycin for 3 days according to multiplicity of infection (MOI). The green fluorescent protein (GFP) expressed in the transfected cells could be detected under the inverted fluorescence microscope. Then overexpression efficiency was confirmed by Western blot. After screened successfully, AGS cells with stable overexpression of USP10 were cultured under standard conditions. Then, cells were collected, and three empty vector samples (EV) and three overexpression samples (OE) were prepared for high-throughput detection. The GV492-USP10 or GV492 were designed by and purchased from Genechem (Shanghai, China). The CDS (2397 bp) of ubiquitin carboxyl-terminal hydrolase 10 isoform 2 (NM_005153.3) was cloned and inserted into the GV492 plasmid to construct GV492-USP10.

### RNA sequencing and expression analysis

After stable overexpression of USP10 in AGS cells, total RNA was extracted with RNAiso Plus (9109, TaKaRa, Shiga, Japan) and chloroform according to the manufacturer’s instructions. After measuring the concentration and purity of the extracted total RNA, the RNA sequencing was conducted by the Beijing Genomics Institute (Shenzhen, China). A detailed description was shown in the Supplementary Materials and Methods. The raw sequence data reported in this paper have been deposited in the Sequence Read Archive (SRA) in the National Center for Biotechnology Information (Maryland, USA) under the BioProject ID PRJNA941161.

### Proteome and ubiquitinome

#### Sample preparation for HPLC-MS/MS

After stable overexpression of USP10 in AGS cells, total protein was extracted. The peptides were separated into 60 fractions with a gradient of 8–32% acetonitrile (pH 9.0) for 60 min by high pH reverse-phase HPLC fractionation using Agilent 300Extend C18 column (5 μm particles, 4.6 mm ID, 250 mm length, Agilent Technologies Inc., USA). Then, 60 fractions were combined into 4 fractions and dried by vacuum freeze for LC-MS/MS analysis. Detailed descriptions could be found in the Supplementary Materials and Methods.

#### Ubiquitinated peptide enrichment

To enrich ubiquitinated peptides, the peptides were dissolved in NETN buffer (100 mM NaCl, 1 mM EDTA, 50 mM Tris-HCl, 0.5% NP-40, pH 8.0) and incubated with pre-washed anti-ubiquitination beads (PTM1104, PTM Bio, Hangzhou, China) at 4 °C overnight with gently shaking. After the beads were washed four times with NETN buffer and twice with deionized water, the bound peptides were eluted with 0.1% trifluoroacetic acid. For LC-MS/MS analysis, the ubiquitinated peptides were desalted with C18 ZipTips (Millipore, Billerica, MA, USA) according to the manufacturer’s instructions.

### HPLC-MS/MS analysis and mass spectrometry data analysis

The peptides were eluted from a ReproSil-Pur basic C18 column (100 μm ID, 1.9 μm particle size, 250 mm length, Dr. Maisch, Tübingen, Germany) and analyzed using an EASY-nLC 1000 UPLC system followed by a Q Exactive Plus mass spectrometer and Orbitrap Fusion mass spectrometer (Thermo Fisher Scientific, Waltham, MA, USA). The MS/MS data were processed using Maxquant software (v1.5.2.8). A detailed description was shown in the Supplementary Materials and Methods.

### Gene expression omnibus (GEO) and the cancer genome atlas (TCGA) datasets analysis

Human gastric adenocarcinoma (GAC) samples: GAC and clinical information, with their corresponding TNFRSF10B expression data, were downloaded from the GEO (http://www.ncbi.nlm.nih.gov/gds/) and TCGA (https://cancergenome.nih.gov/) databases. The TNFRSF10B mRNA expression level in GSE84437 was obtained from the intensity of probe ILMN_2331010, and for survival analysis (433 cases with overall survival). The Xena Functional Genomics Explorer (https://xenabrowser.net/) was used to download TNFRSF10B expression data and perform survival analysis (295 cases with overall survival).

### Tissue sample and data collection

#### Inclusion criteria

From February 2014 to April 2017, consecutive cases of GC identified by the Departments of Gastrointestinal Surgery and Pathology, as well as through cancer registries at Renmin Hospital of Wuhan University, that met the following criteria were included in the study: GC patients had not received radiotherapy or chemotherapy, or other related tumor treatments prior to surgery. The histological type of GC has been confirmed by pathologists as GAC. GC patients had complete clinicopathological data.

#### Exclusion criteria

GC cases for which tissue samples were taken from patients who underwent endoscopic submucosal dissection or endoscopic biopsy, and those GC patients who died within two weeks after surgery were excluded.

Finally, 194 cases of GAC patients were enrolled. Clinical pathological data of these patients were collected from the medical records in the hospitals, including age, sex, tumor size, tumor differentiation, histological type, Lauren classification, depth of invasion, lymph node metastasis, and TNM stage. In this study, the TNM stage was confirmed according to the Union for International Cancer Control (UICC) classification (8th edition). For immunohistochemical staining analysis, 194 formalin-fixed, paraffin-embedded GAC tumor tissues were selected. Then, the tissue microarrays were made. For survival analysis, the 194 cases were followed up, and the follow-up time was terminated in August 2020. The survival status of 171 cases were acquired from the clinic records and through patient or family contacts, 23 cases were excluded due to the missing of follow-up. The duration of overall survival was recorded as the time interval from the date of surgical resection to the date of death or the last known date alive.

### Immunohistochemical staining

Immunohistochemical staining was performed according to the standard protocol. Detailed descriptions could be found in the Supplementary Materials and Methods. The immunohistochemical staining results of TNFRSF10B in all sections were assessed by two independent pathologists (Zeng and Zhang) who were uninformed about the diagnosis outcome. Protein expression levels were confirmed according to the average score of the two pathologists’ evaluations. In our study, the immuno-staining score (ISS) of TNFRSF10B was used to elucidate its protein expression levels according to the following formula: ISS = PS × IS, where PS: percentage score represented the percentage of immuno-positive cells, which was graded as 0 (< 10%), 1 (10–25%), 2 (26–50%), 3 (51-75%), and 4 (> 75%); IS: intensity score represented the intensity of immuno-staining, which was determined as 0 (absent), 1 (weak staining), 2 (moderate staining), and 3 (strong staining). After immuno-staining analysis, TNFRSF10B protein expression levels were divided into two groups based on the ISS: low expression group (ISS ≤ 6) and high expression group (ISS > 6).

### Animal experiments

The metastatic tumor model was constructed as previously described by Hamaidi et al. [22]. AGS cells were divided into two groups and transfected with si-USP10 or si-NC, respectively, and MKN45 cells were divided into three groups and transfected with si-USP10, si-TNFRSF10B, or si-NC, respectively. A detailed description was shown in the Supplementary Materials and Methods.

### Statistical analysis

Statistical analyses were performed using GraphPad Prism 9.0 (GraphPad Software, San Diego, CA, USA). All quantitative data are presented as mean ± SD. Statistical significance was analyzed by one-way analysis of variance (ANOVA) with Bonferroni’s multiple comparison test. The difference of TNFRSF10B positive expression rate in the various groups of human tissues was analyzed using the Chi-Square test. The possible relationship between TNFRSF10B expression and the clinical pathological features was analyzed by Spearman’s test. For survival analysis, the survival curves were calculated using the Kaplan-Meier method and compared by the log-rank test. *p* value < 0.05 was regarded as statistically significant.

### Ethics statement

All animal experiments were approved by the Laboratory Animal Ethics Committee of Renmin Hospital of Wuhan University (WDRM-20230205A) and performed according to the guidelines of animal research. The clinical sample study was approved by the Clinical Research Ethics Committee, Renmin Hospital of Wuhan University (WDRY2022-K162). We obtained the application for exemption of informed consent for the clinical sample study.

## Results

### Identification of the potential direct substrates of USP10

**Fig. 1:**
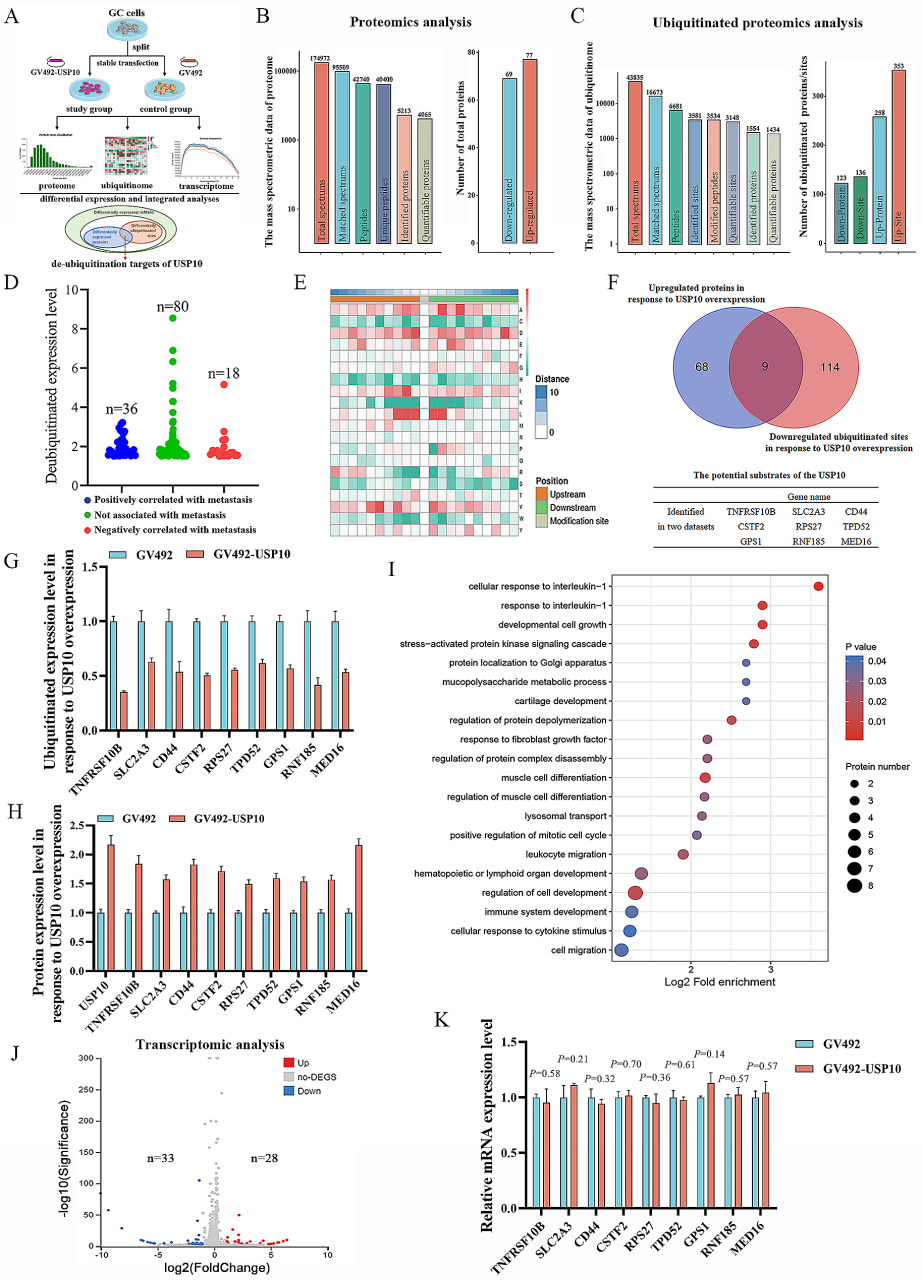
The proteomics analysis, ubiquitinated proteomics analysis, and transcriptomic analysis for identifying potential USP10 substrates. **A** The flow diagram of the high-throughput strategy for screening de-ubiquitination targets of USP10 in AGS cells. **B** The result of proteomics analysis. **C** The result of ubiquitinated proteomics analysis. **D** Proteins deubiquitinated by USP10 were classified into three categories. The blue dots represent deubiquitinated proteins that are positively correlated with GC metastasis. The red dots represent deubiquitinated proteins that are negatively correlated with GC metastasis. The green dots represent deubiquitinated proteins that are not correlated with GC metastasis. **E** Thermal map of motif analysis of amino acids near the ubiquitin sites. Red indicates a significant increase in the probability of the amino acid occurring near the modification site. Green indicates a significant decrease in the probability of the amino acid occurring near the modification site. **F** Venn diagram showed the number of overlapping proteins in the two datasets, and potential substrates of the USP10. **G** The ubiquitinated expression level of 9 potential substrates of USP10 in response to USP10 overexpression. **H** The protein expression level of 9 potential substrates of USP10 in response to USP10 overexpression. **I** GO Biological Process enrichment analysis of USP10 upregulating proteins in proteomics analysis. **J** Volcanic plot showed differentially expressed genes. **K** The transcriptomic analysis revealed the mRNA expression level of potential substrates of the USP10.

In order to systematically identify the de-ubiquitination targets of USP10 in GC cells, we constructed an AGS cell line with stable overexpression of USP10 by transfected the GV492-USP10, and then established a high-throughput strategy including proteome, ubiquitinome and transcriptome analyses between the study group (GV492-USP10) and the control group (GV492) (Fig. 1A). During this process, the mass spectrometry-based TMT/iTRAQ Labeling with peptides quantitative proteomics analysis was performed. A total of 5213 proteins were identified, of which 4065 were quantifiable proteins (Fig. 1B). Subsequently, we used a criterion of 1.5-fold change or greater between these two groups as differential protein candidates. The results showed that 77 upregulated proteins and 69 downregulated proteins were identified in AGS cells after overexpression of USP10 (Fig. 1B).

To further characterize the de-ubiquitination substrates of USP10, we employed an affinity-based ubiquitinated peptide enrichment approach to systematically quantify the change of ubiquitinome in AGS cells with USP10 overexpression. As the tryptic peptides with TMT/iTRAQ Labeling were fractionated into fractions by high pH reverse-phase HPLC, we carried out affinity enrichment of ubiquitin modified peptides using pan antibody-based PTM enrichment. The enriched peptides were then subjected to LC-MS/MS analysis for ubiquitinome quantification. We identified 3581 ubiquitination sites on 1554 proteins, of which 3148 sites on 1434 proteins were quantifiable (Fig. 1C). With a criterion of ≥ 1.5-fold change between the study group and the control group, we finally obtained 136 significant downregulated ubiquitin sites on 123 proteins (Fig. 1C), which might directly be regulated by USP10. We used UALCAN website (https://ualcan.path.uab.edu/analysis.html) to conveniently calculate and define GC progression related genes according to gene expression and clinical samples data of TCGA (STAD). Based on their relationship with GC progression related genes, proteins deubiquitinated by USP10 can be classified into three categories (Table S3). Among them, 36 deubiquitinated sites on 29 deubiquitinated proteins could be associated with promotion metastasis, and 18 deubiquitinated sites on 17 deubiquitinated proteins could be associated with inhibiting metastasis (Fig. 1D, Table S3). To investigate whether there was any particular amino acid preference adjacent to the ubiquitinated sites, we analyzed the model of sequences constituted with amino acids near the ubiquitin sites in all protein sequences using Soft MoMo. The results showed that aspartic acid (D) and leucine (L) were notably close to the ubiquitin sites, whereas lysine (K) and arginine (R) were less preferred near the ubiquitin sites (Fig. 1E), and the motif was xxxxxxxxLx_K_xxxxxxxxxx (Motif Score = 8.69) or xxxxxxxLxx_K_xxxxxxxxxx (Motif Score = 7.37) (Fig. S1A).

To determine the direct substrates of USP10, we compared the upregulated proteins and downregulated ubiquitinated sites. Ideally, the direct substrates of USP10 were identified from two independent experiments (i.e., proteome and ubiquitinome). We considered that a protein identified from two datasets could be a potential substrate of USP10. Using this criterion, we identified 9 potential substrates of USP10, including TNFRSF10B, SLC2A3, CD44, CSTF2, RPS27, TPD52, GPS1, RNF185, and MED16 (Fig. 1F). The ubiquitinated expression levels of these 9 proteins were downregulated, while the total protein expression levels were upregulated in response to USP10 overexpression (Fig. 1G and H). Signal pathway enrichment analysis demonstrated that USP10 upregulating proteins were enriched in EMT-related GO functions and pathways, such as cell migration, epithelial cell-cell adhesion, extracellular matrix, pathways in cancer, etc. (Fig. 1I, Fig. S1B-D). Moreover, 61 differentially expressed genes were identified by transcriptome sequencing (Fig. 1J). However, none of these genes and their signal pathways overlapped with the 9 potential substrates of USP10 (Fig. 1K, Fig. S2), further indicating that these proteins were upregulated by USP10 at the level of translation or post-translational modifications. The motif analysis showed that the modified sequence of CD44 and TPD52 belonged to xxxxxxxLxx_K_xxxxxxxxxx, and the modified sequence of RPS27 and MED16 belonged to xxxxxxxxLx_K_xxxxxxxxxx.

### USP10 regulates the migration and invasion of GC cells

**Fig. 2:**
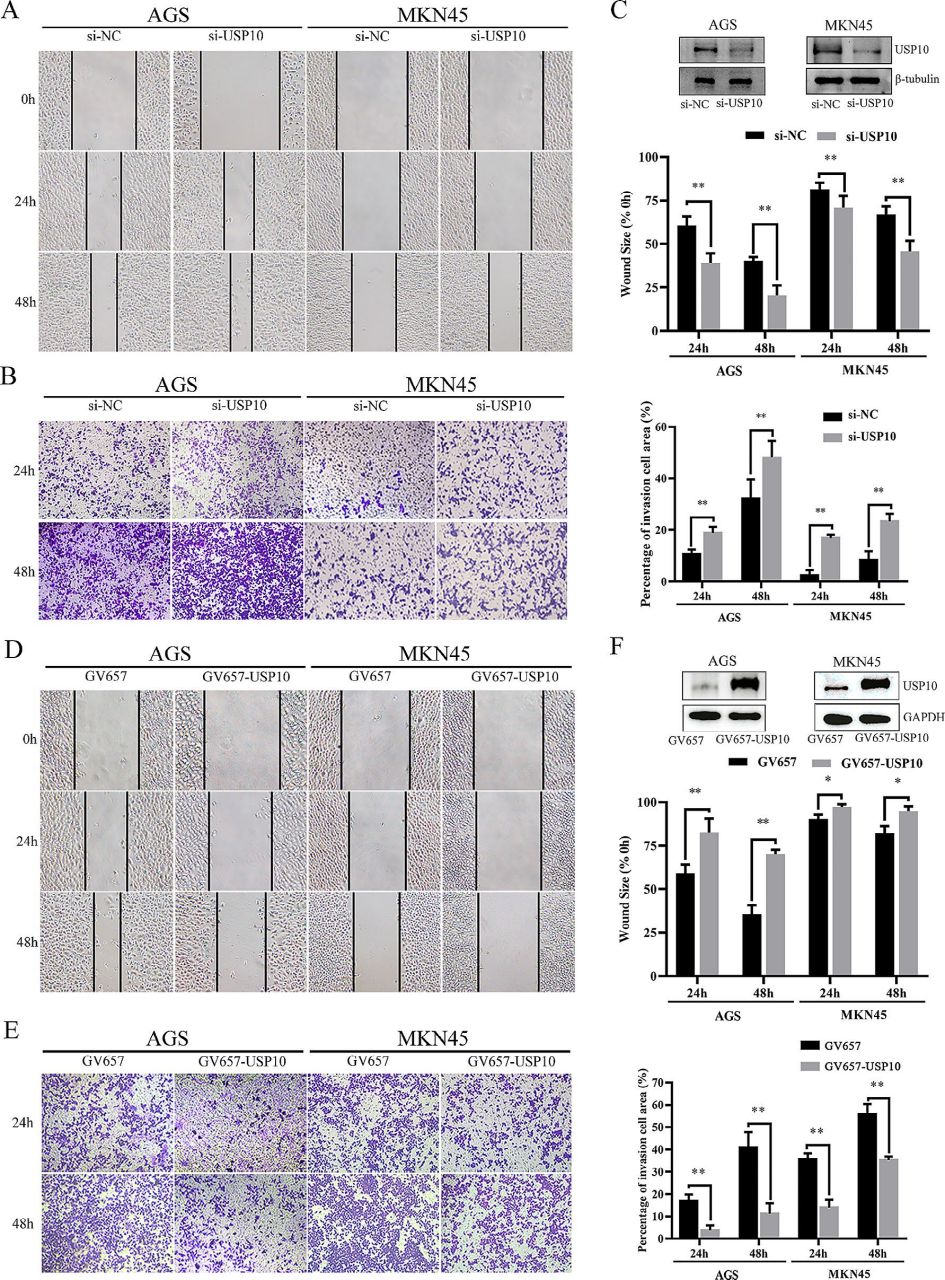
USP10 regulates the migration and invasion of GC cells. **A** Wound-healing assay (left) and quantitative analysis (right) in human GC cell lines (AGS or MKN45) transfected with si-USP10, original magnification, ×40. **B** Transwell invasion assay (left) and quantitative analysis (right) in human GC cell lines transfected with si-USP10, original magnification, ×40. **C** The protein level of USP10 was measured when si-USP10 was transfected into AGS cells or MKN45 cells. **D** Wound-healing assay (left) and quantitative analysis (right) in human GC cell lines transfected with GV657-USP10, original magnification, ×40. **E** Transwell invasion assay (left) and quantitative analysis (right) in human GC cell lines transfected with GV657-USP10, original magnification, ×40. **F** The protein level of USP10 was measured when GV657-USP10 was transfected into AGS cells or MKN45 cells. *n* = 6 per group, *p < 0.05, **p < 0.01

Three siRNAs targeted USP10 mRNA were designed and transfected to AGS or MKN45 cells. Among them, the si-USP10-2 knockdown effect was the most stable (Fig. S3). Then, the result showed that down-regulation of USP10 notably enhanced the ability of migration compared with the negative control group (si-NC) (Fig. 2A). Meanwhile, the invasive cells were observably increased in the GC cells after USP10 knockdown (Fig. 2B). USP10 protein levels were markedly reduced when si-USP10 was transfected into AGS or MKN45 cells (Fig. 2C). Furthermore, to exclude the off-target effect of siRNA, three si-USP10s (40 nM) were transfected respectively. The results of wound healing showed that all these si-USP10s increased the migration ability of AGS cells (Fig. S4A). Surprisingly, when the concentration of si-USP10s increased to 60 nM, the effect of si-USP10s on cell migration was reversed (Fig. S4B).

To further verify that USP10 inhibited the migration and invasion of GC cells, we constructed overexpressed plasmid GV657-USP10. Figure 2D showed that up-regulation of USP10 significantly reduced the ability of migration compared to the negative control group. Meanwhile, the invasive cells were decreased in the group of GV657-USP10 transfected after 24 h and 48 h (Fig. 2E). USP10 protein levels were markedly increased when the GV657-USP10 was transfected into AGS or MKN45 cells (Fig. 2F).

### USP10 regulates TNFRSF10B stabilization via de-ubiquitination in GC cells

**Fig. 3:**
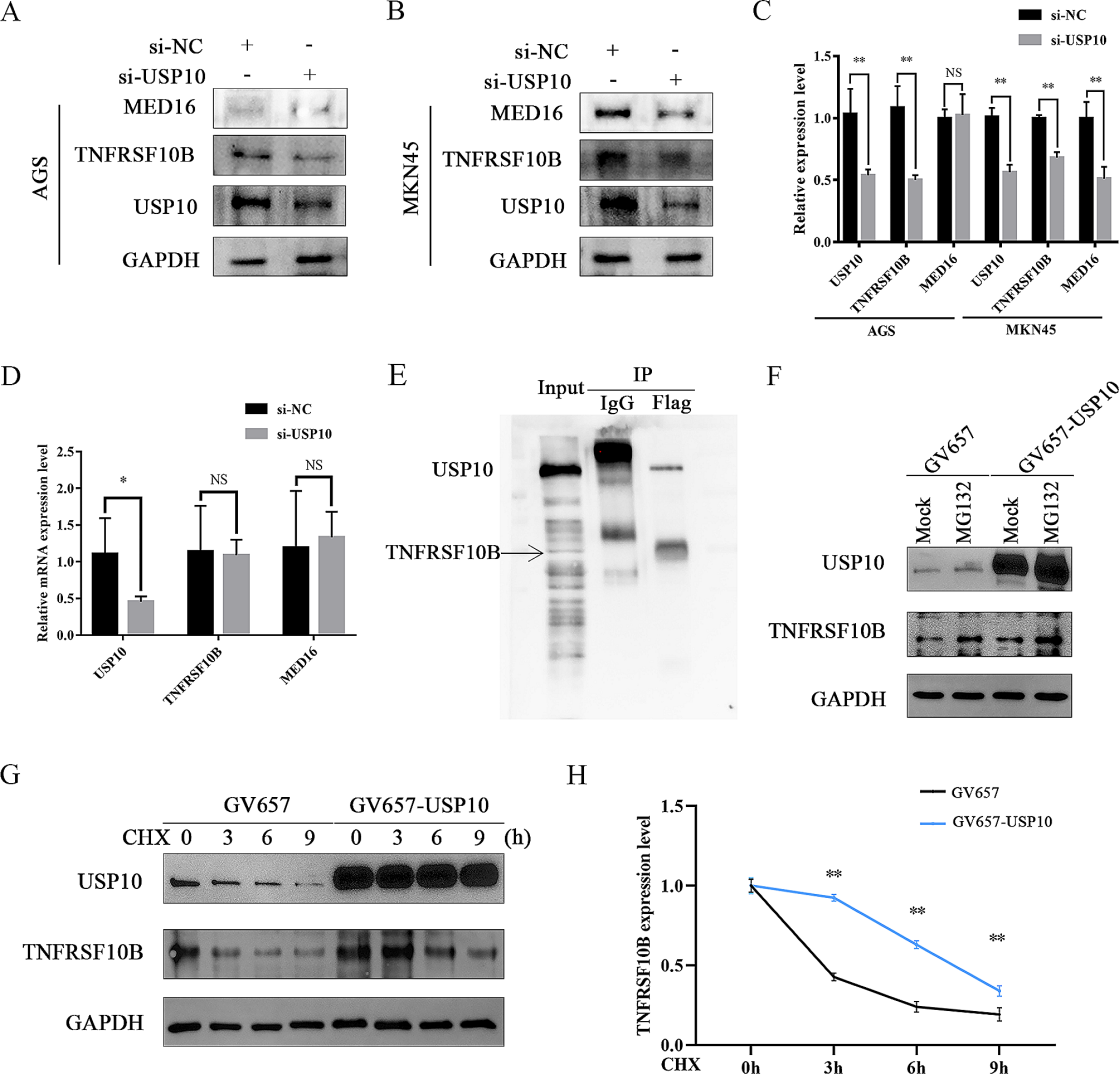
USP10 regulated TNFRSF10B stabilization via de-ubiquitination in GC cells. **A**, **B** and **C** The protein expression level of USP10, TNFRSF10B, and MED16 were measured by WB after si-USP10 transfected into AGS cells or MKN45 cells. **D** The mRNA expression level of USP10, TNFRSF10B, and MED16 were measured by qRT-PCR after si-USP10 transfected into MKN45 cells. **E** Co-IP assay: after 293T cells were transfected with Flag-GV657-USP10 plasmid (Flag tag), the cell lysates were co-immunoprecipitated by anti-Flag (USP10) antibody with IgG as negative control. The immunoprecipitated proteins of USP10 and TNFRSF10B were detected by WB, that is, the membrane was incubated with TNFRSF10B antibody. Then, the membrane was incubated with anti-Flag (USP10) antibody. **F** After 20 µM MG132 treated 8 h, the protein expression level of USP10 and TNFRSF10B were measured by WB in 293T cells transfected with GV657-USP10. **G**, **H** After 10 µM cycloheximide (CHX) treated at different time points, the protein expression level of USP10 and TNFRSF10B were measured in 293T cells transfected with GV657-USP10, WB analysis (left) and densitometry analysis (right). *n* = 6 per group, *p < 0.05, **p < 0.01

Herein, we chose TNFRSF10B and MED16 as the representatives for further verification. After transfection with si-USP10, the protein expression levels of TNFRSF10B were reduced in AGS and MKN45 cells, while the protein expression levels of MED16 were only reduced in MKN45 cells (Fig. 3A-C). In addition, the mRNA expression level of TNFRSF10B and MED16 did not change significantly after si-USP10 transfected into MKN45 cells (Fig. 3D). The Co-IP assays showed that USP10 could bind to TNFRSF10B in 293T cells transfected with GV657-USP10 (Fig. 3E), further indicating that USP10 can regulate the de-ubiquitination of TNFRSF10B. After treatment with the proteasome inhibitor MG132, TNFRSF10B protein expression was significantly increased in the GV657-USP10 group (Fig. 3F). And the cycloheximide (CHX, a protein synthesis inhibitor) assays showed that the degradation rate of TNFRSF10B protein at different time points (0, 3, 6, and 9 h) slowed down significantly in the GV657-USP10 group (Fig. 3G and H), further confirming that USP10 directly upregulated TNFRSF10B protein expression via de-ubiquitination.

### Downregulated TNFRSF10B enhances the migration ability of GC cells in vitro

**Fig. 4:**
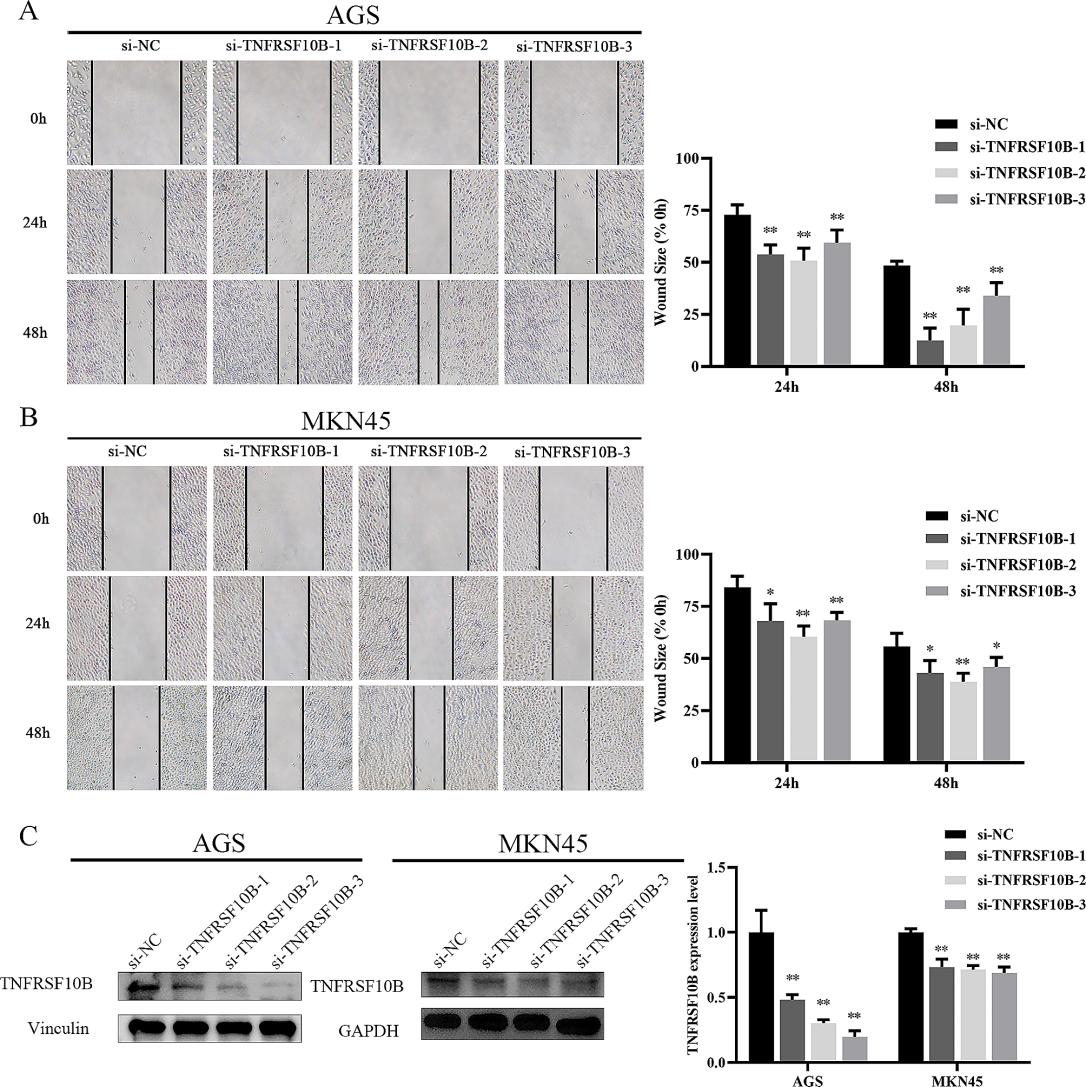
Downregulated TNFRSF10B enhanced the migration ability of GC cells in vitro. **A**, **B** Wound-healing assay (left) and quantitative analysis (right) in human GC cell lines (AGS or MKN45) transfected with si-TNFRSF10B-1, si-TNFRSF10B-2, or si-TNFRSF10B-3, original magnification, ×40. **C** The protein level of TNFRSF10B was measured when si-TNFRSF10B-1, si-TNFRSF10B-2, or si-TNFRSF10B-3 was transfected into AGS cells or MKN45 cells, WB analysis (left) and densitometry analysis (right). *n* = 6 per group, *p < 0.05, **p < 0.01

GO enrichment analysis based on proteomics analysis result showed that USP10 upregulating TNFRSF10B involvement in the cell migration signaling pathway of AGS cells (Table S4). Therefore, we further assessed the migration ability of AGS or MKN45 cells which were transfected with si-TNFRSF10B-1, si-TNFRSF10B-2, or si-TNFRSF10B-3 by the wound-healing experiments. After si-TNFRSF10B-1, si-TNFRSF10B-2, or si-TNFRSF10B-3 transfected into AGS or MKN45 cells, the migration ability of GC cells was notably elevated (Fig. 4A and B). Western blot assays demonstrated that TNFRSF10B protein levels were markedly reduced when si-TNFRSF10B-1, si-TNFRSF10B-2, or si-TNFRSF10B-3 was transfected into AGS or MKN45 cells (Fig. 4C).

### Downregulated USP10 or TNFRSF10B promotes GC metastasis in vivo

**Fig. 5:**
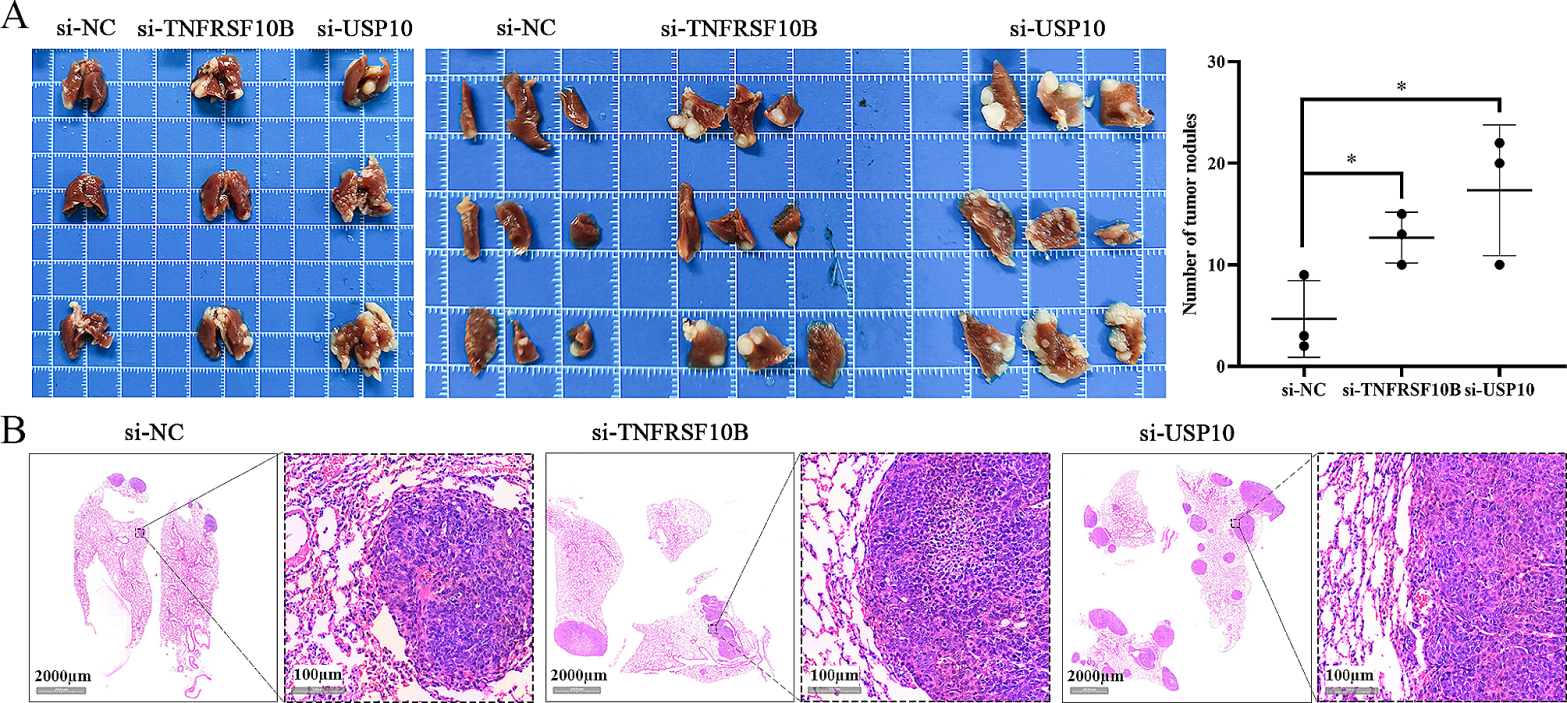
Downregulated USP10 or TNFRSF10B promotes GC lung metastasis in vivo. **A** Lung tissues were examined when mice were euthanized after MKN45 cells transfected with si-USP10 or si-TNFRSF10B were intravenously injected for 5 weeks (*n* = 5 per group, tumor formation rate = 60%), as well as the numbers of tumor nodules were counted and statistical analysis was performed. **B** The H&E staining in lung tissue sections. *p < 0.05

In the metastatic tumor models, the MKN45 cells transfected with si-USP10, si-TNFRSF10B, or si-NC were intravenously injected into nude mice via the tail vein. After 5 weeks, the mice were euthanized, and lung metastatic nodules were observed in 3 out of 5 nude mice in each group. Compared with the si-NC group, the si-USP10 group or si-TNFRSF10B group had significantly more and larger lung metastatic nodules (Fig. 5). In addition, the AGS cells transfected with si-USP10 or si-NC were also intravenously injected into nude mice. Although 3 out of 5 nude mice in each group observed liver metastatic nodules instead of lung metastatic nodules, the number of liver metastatic nodules in the si-USP10 group was significantly higher than that in the si-NC group (Fig. S5). These data revealed that downregulated USP10 or TNFRSF10B promoted the metastatic ability of GC cells in vivo.

### USP10 suppresses EMT via stabilizing TNFRSF10B expression

**Fig. 6:**
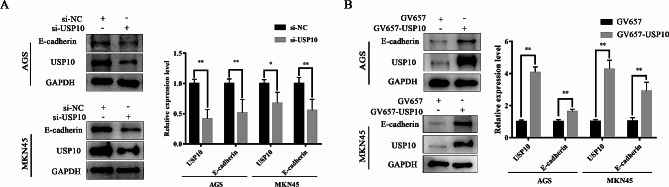
USP10 regulated E-cadherin expression in GC cells. **A**, **B** The E-cadherin expression was assessed by WB analysis. *n* = 6 per group, *p < 0.05, **p < 0.01

EMT can confer the migration and invasion ability of cancer cells [23], and we have previously reported that the expression of USP10 was positively correlated with the expression of E-cadherin in a large number of clinical samples [12]. Therefore, we detected the expression of E-cadherin, one of the markers of EMT, in AGS or MKN45 cells after USP10 knockdown or overexpression. In this study, we found that the protein expression levels of E-cadherin were downregulated in the group of si-USP10, and were upregulated in the group of GV657-USP10 (Fig. 6). The results demonstrated that downregulated USP10 induced EMT by regulating E-cadherin.

**Fig. 7:**
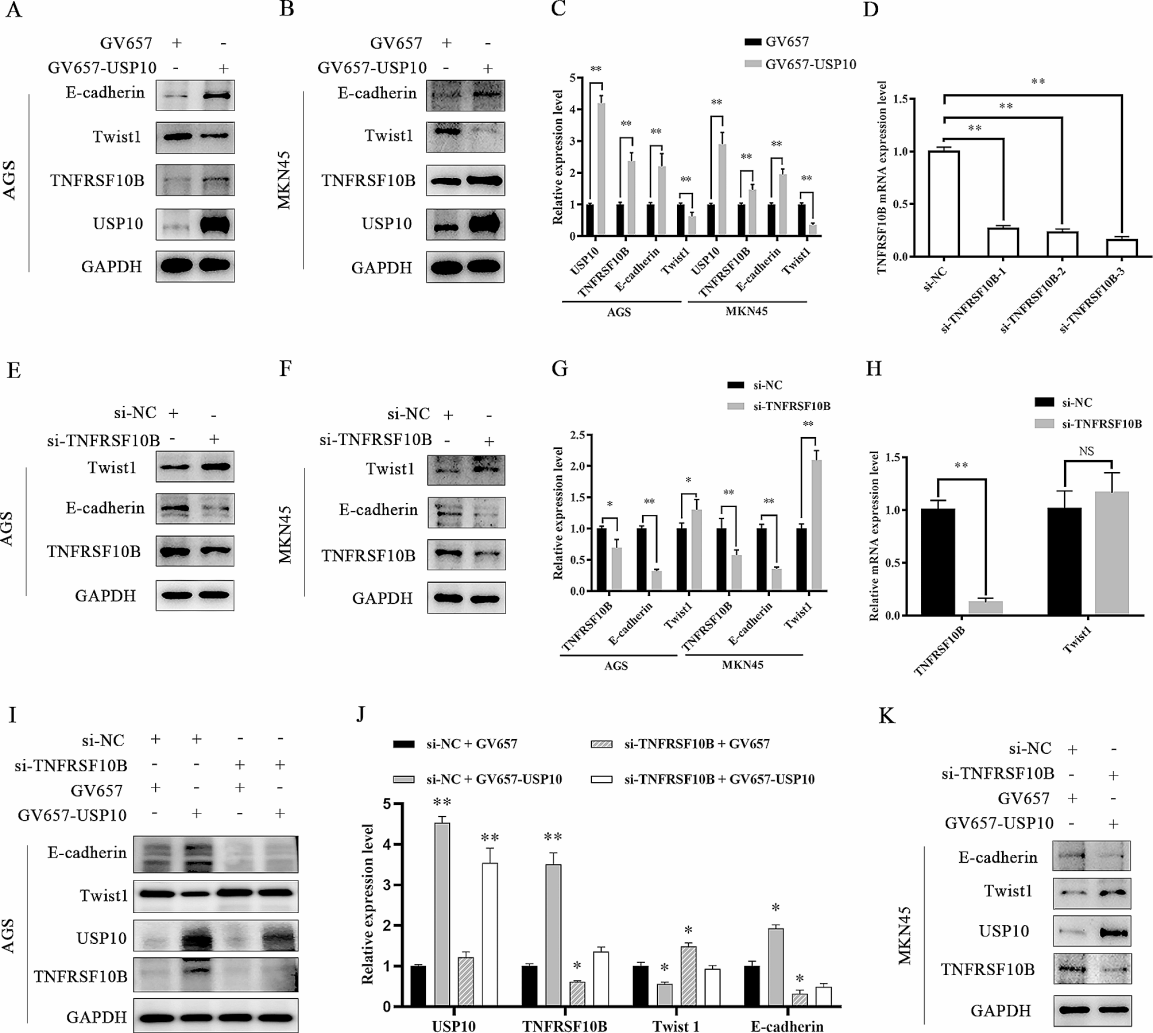
USP10 suppressed EMT through stabilizing TNFRSF10B protein in GC cells. **A**, **B** and **C** The protein expression level of USP10, TNFRSF10B, Twist1, and E-cadherin were measured by WB after GV657-USP10 transfected into AGS cells or MKN45 cells. **D** The knockdown efficiency of si-TNFRSF10B-1, si-TNFRSF10B-2, or si-TNFRSF10B-3 was assessed by qRT-PCR. **E**, **F** and **G** The protein expression level of TNFRSF10B, Twist1, and E-cadherin were measured by WB after si-TNFRSF10B transfected into AGS cells or MKN45 cells. **H** The mRNA expression level of TNFRSF10B and Twist1 were measured by qRT-PCR after si-TNFRSF10B transfected into MKN45 cells. *n* = 6 per group, *p < 0.05, **p < 0.01. **I**, **J** and **K** The protein expression level of USP10, TNFRSF10B, Twist1, and E-cadherin were measured by WB after GV657-USP10 and si-TNFRSF10B transfected into AGS cells or MKN45 cells, *n* = 6 per group, *p < 0.05, **p < 0.01, vs. si-NC + GV657.

Figure 7A-C showed that the expressions of TNFRSF10B and E-cadherin were upregulated, while Twist1 expression was downregulated after GV657-USP10 transfected into AGS or MKN45 cells. Twist1 is one of the most important transcription factors, which negatively regulates E-cadherin [24, 25]. Thus, we speculated that USP10/TNFRSF10B may regulate the EMT of GC cells through the Twist1-E-cadherin pathway. The qRT-PCR assay demonstrated that the knockdown efficiency of si-TNFRSF10B-1/2/3 was very high, and si-TNFRSF10B-2 was used for follow-up experiments (Fig. 7D). E-cadherin expression was reduced, as well as Twist1 expression was increased after si-TNFRSF10B was transfected into AGS or MKN45 cells (Fig. 7E-G). Whereas, the mRNA expression level of Twist1 did not change significantly, indicating that TNFRSF10B and USP10 regulated Twist1 at the protein level (Fig. 7H). Furthermore, we found that USP10 overexpression after knock-down of TNFRSF10B in AGS or MKN45 cell lines, USP10 would be deprived of the ability for inhibiting Twist1 expression and upregulating E-cadherin expression (Fig. 7I-K). Thus, we believed that USP10 suppressed EMT through upregulating TNFRSF10B expression in GC cells.

### TNFRSF10B expression in human GC tissues

**Fig. 8:**
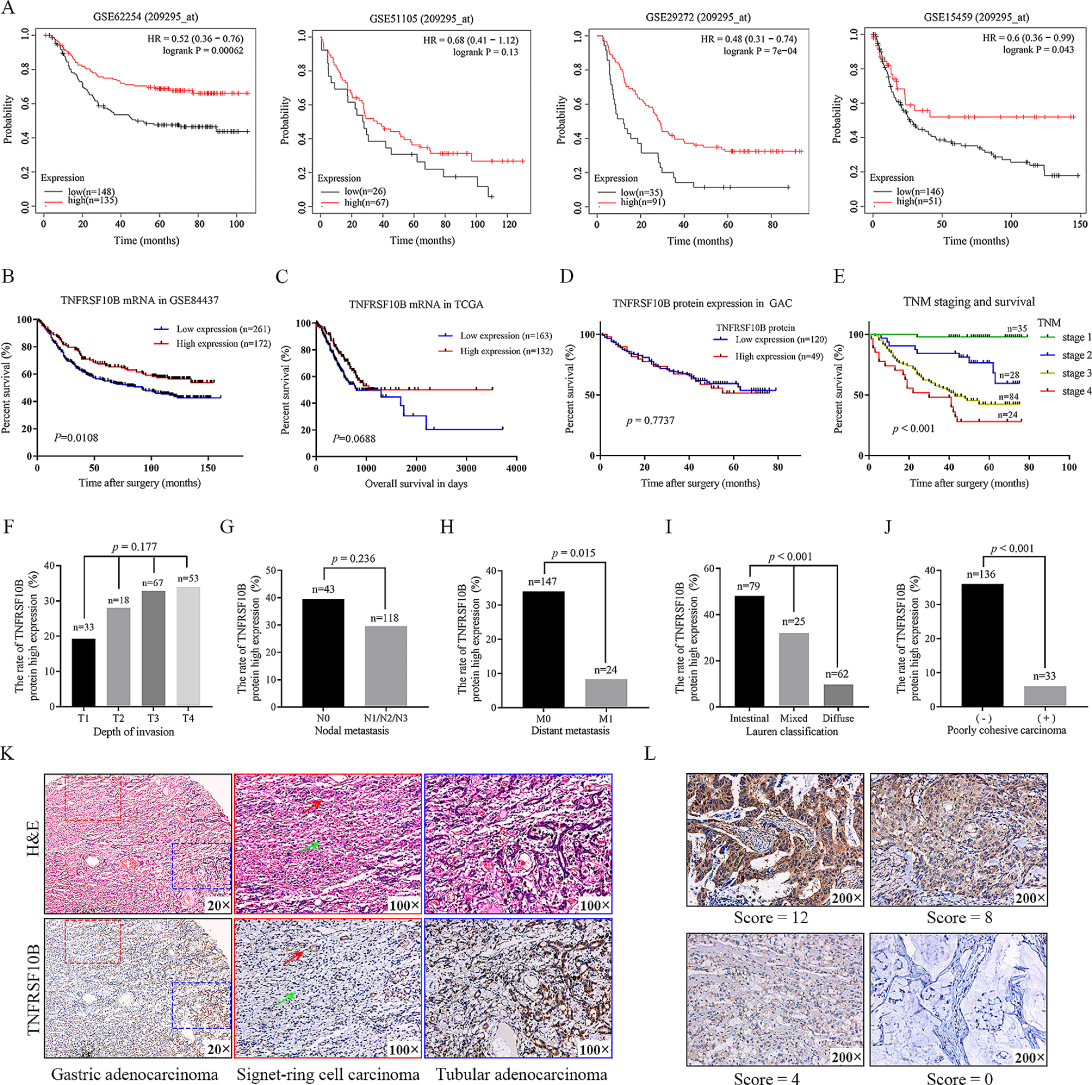
TNFRSF10B expression in human GC tissues. **A** The Kaplan-Meier overall survival analysis of TNFRSF10B in four GEO datasets of GC (https://kmplot.com/ analysis). **B** The relationship of TNFRSF10B mRNA expression to the overall survival of GAC patients in GSE84437 (*p* < 0.05). **C** TCGA datasets showed the relationship of TNFRSF10B mRNA expression to the overall survival of GAC patients (*p* > 0.05). **D** The relationship of TNFRSF10B protein expression to the overall survival of GAC patients in our cohort (*p* > 0.05). **E** The relationship of TNM stage to the overall survival of GAC patients in our cohort (*p* < 0.001). **F** The high expression rate of TNFRSF10B protein in depth of invasion T1, T2, T3, and T4 GAC (*p* > 0.05, Chi-square test for trend). **G** The high expression rate of TNFRSF10B protein in nodal metastasis N0 and N1/N2/N3 GAC (*p* > 0.05, Chi-square test for trend). **H** The high expression rate of TNFRSF10B protein in distant metastasis M0 and M1 GAC (*p* < 0.05, Chi-square test). **I** The high expression rate of TNFRSF10B protein in Lauren’s classification of intestinal type, mixed type, and diffuse type GAC (*p* < 0.001, Chi-square test). **J** The high expression rate of TNFRSF10B protein in poorly cohesive carcinoma and other adenocarcinoma GAC (*p* < 0.001, Chi-square test). **K** The H&E and IHC staining in the signet-ring cell carcinoma and tubular adenocarcinoma of paraffin-embedded GAC, green arrow: signet-ring cell carcinoma, red arrow: residual mucosal glands, original magnification, ×20, ×100. **L** The representative graph for immuno-staining score of TNFRSF10B, original magnification, ×200

To investigate the correlation between TNFRSF10B mRNA expression and the prognosis of GAC patients, 433 samples in GSE84437 and 295 TCGA (STAD) samples were respectively used for survival analysis, and we used the Kaplan-Meier plotter (https://kmplot.com/analysis) including GEO, EGA, and TCGA databases to obtain more cohorts for survival analysis. Except for GSE51105, the GSE62254, GSE29272, GSE15459, and GSE84437 datasets analyses showed that TNFRSF10B mRNA expression was significantly positively correlated with the GAC patient overall survival (Fig. 8A and B). In addition, the results of the GSE62254 and GSE15459 datasets analysis demonstrated that the TNFRSF10B mRNA expression was significantly positively correlated with the first progression of GAC patients (Fig. S6A). Moreover, TNFRSF10B mRNA expression was significantly positively associated with the post progression survival of GAC patient in GSE62254 samples, while TNFRSF10B mRNA expression was not associated with the post progression survival of GAC patient in GSE15459 samples (Fig. S6B). However, TNFRSF10B mRNA expression was not associated with the overall survival of GAC patient in TCGA (STAD) samples (Fig. 8C). In our GAC cohort (135 males and 59 females; age range, 22–75 years old; mean age 59.7 ± 10.2 years old) study, we found that the TNFRSF10B protein expression was also not associated with the overall survival of GAC patient (Fig. 8D). Kaplan-Meier analysis showed that the decreased GAC patient survival was only significantly correlated with later TNM stage, one of the well-established prognostic factors (Fig. 8E), which demonstrated the representativeness of our GAC cohort. Next, the relationship between TNFRSF10B protein expression and the TNM stage of GAC was further analyzed in our 171 patients. The results showed that the expression rate of TNFRSF10B protein in the group without distant metastasis (M0) was significantly higher than that in the group with distant metastasis (M1), while no statistical difference was observed in T stage and N stage (Fig. 8F-H). Moreover, the expression trends between the T stage and the N stage or the M stage were opposite, which could partially explain why TNFRSF10B protein expression was not related to the survival of patients in our GAC cohort. Finally, we found that the expression rates of TNFRSF10B protein in the diffuse type GAC and poorly cohesive carcinoma were lower than that in the intestinal type GAC and non-poorly cohesive carcinoma, respectively (Fig. 8I and J), indicating that TNFRSF10B was involved in the regulation of GC cell adhesion. Representative histological features, IHC staining specimens, and scoring criteria were presented in Fig. 8K and L.

## Discussion

In our previous study, we reported a significant negative correlation between USP10 expression and deeper gastric wall invasion, positive lymph node status, and late stage TNM staging in GC tissue samples. Meanwhile, USP10 protein was identified as an independent risk factor for poor overall survival in GC patients [12]. However, it remains unclear whether downregulation of USP10 expression is the cause or the result of GC progression. In the present study, the experimental results obtained from in vitro cell models showed that downregulated USP10 significantly promoted GC cell migration and invasion, while upregulated USP10 significantly inhibited GC cell migration and invasion. These findings support the conclusion drawn from our previous clinical research on USP10 in GC [12], and indicate that USP10 plays a role as a tumor metastasis suppressor in the progression of GC. In addition, similar to our conclusion, Cheng et al. found that USP10 acts as a tumor suppressor gene by regulating the deubiquitination of wild-type p53 protein in GC cells [13]. However, some studies have come to the opposite conclusion, reporting that the de-ubiquitination of RFC2 or DDX21 by USP10 leads to the promotion of GC metastasis [10, 11]. In our study, we also observed an interesting phenomenon that the effect of si-USP10s on cell migration was reversed when the concentration of si-USP10s was adjusted from low dose (40 nm) to high dose (60 nm). Therefore, we believed that the extent of USP10 protein knockdown might be the key factor in determining the biological behavior of GC, which might partially explain why opposite conclusions were drawn from different studies.

After overexpression of USP10 in GC cells, 146 proteins and 489 ubiquitin sites exhibited differential expression through proteome and ubiquitinome analyses. Among them, 136 significant downregulated ubiquitin sites on 123 proteins were obtained, which were likely to be directly regulated by USP10. However, RFC2 and DDX21, reported as de-ubiquitination targets for USP10 in GC cell migration [10, 11], did not appear in our screening list. Therefore, we speculated that different expression levels of USP10 might exert opposite biological functions by deubiquitinating different protein substrates. In addition, after comparing with the GC metastasis related genes defined by TCGA-STAD clinical samples detection, the proteome and ubiquitinome results showed that 36 deubiquitinated sites regulated by USP10 were associated with promotion metastasis, and 18 deubiquitinated sites were associated with inhibiting metastasis. So, we believed that the ultimate biological behavior of GC cells regulated by USP10 was the result of the cumulative effects of the multiple proteins corresponding to the above deubiquitinated sites.

To further clarify the role of USP10 in the progression of GC, we constructed an in vivo GC metastasis model. The results showed that downregulated USP10 in vivo significantly promoted the metastatic ability of GC cells, including liver metastasis (AGS cells) and lung metastasis (MKN45 cells). These findings further supported the results we obtained from in vitro cell models and confirmed that USP10 plays a role as a tumor metastasis suppressor in the progression of GC. The minor flaw in our experiment was that the AGS cells injected into the tail veins of nude mice only resulted in liver metastasis, while the MKN45 cells injected into the tail veins of nude mice only resulted in lung metastasis. However, in other studies, intravenous injection of AGS or MKN45 cells injected into nude mice could initiate lung and liver metastasis [26, 27].

Ideally, the direct substrate of USP10 needs to meet three conditions: upregulation of total protein expression, downregulation of ubiquitination sites, and no significant changes in mRNA. According to this standard, we screened 9 potential substrates of USP10, which were TNFRSF10B, SLC2A3, CD44, CSTF2, RPS27, TPD52, GPS1, RNF185, and MED16. Among them, TNFRSF10B, is a member of the tumor necrosis factor superfamily, and contains the cytoplasmic death domain, thus it can transduce apoptotic signals in many cancers [28, 29], including GC [30–32]. In an experimental pulmonary metastasis model, TNFRSF10B agonist MEDI3039 can significantly inhibit lung metastasis of triple-negative breast cancer cells [33]. Oh et al. found that suppression of TNFRSF10B induced recruitment of FADD, caspase-8, and SphK1/S1P to stabilize TRAF2, followed by activation of the ERK and JNK/AP-1 signaling pathway to upregulate MMP1 expression, resulted in promoting cancer cell invasion and metastasis [34, 35]. Trail-r (Tnfrsf10b)^–/–^ mice showed increasing lymph node metastasis of squamous cell carcinoma without influencing primary tumor progression [36]. Therefore, we chose TNFRSF10B as a representative for further verification. The Co-IP, protein stabilization, and wound-healing assays showed that USP10 directly interacted with TNFRSF10B to stabilize it, and then inhibited GC cell migration in vitro. Furthermore, the results of the metastatic tumor model also showed that downregulated TNFRSF10B in vivo promoted the metastatic ability of GC cells. The encoded protein of TNFRSF10B consists of approximately 440 amino acids. The ubiquitinomic and motif analysis showed that the de-ubiquitination site (lysine, K) regulated by USP10 is located at position 245 of the TNFRSF10B protein, and the modified amino acid sequence is VLPYL_K_GICSGGGGDPER. Further researches are needed to confirm whether mutating this de-ubiquitination site or modified sequence can induce the migration and invasion of GC cells.

EMT is a key program in cancer cell invasion and metastasis [14, 15]. GO and KEGG pathway enrichment analysis demonstrated that proteins upregulated by USP10 in GC were enriched in signaling pathways related to EMT. Therefore, we further detected the relationship between USP10/ TNFRSF10B and the major hallmarks of EMT (such as E-cadherin and Twist1) in GC cells. In our study, after USP10 was knocked down, the expression levels of E-cadherin in GC cells were downregulated, and the expression of transcription factor Twist1 which is a negative regulator of E-cadherin was activated. On the contrary, after USP10 was overexpressed, the expression of E-cadherin was enhanced. These results suggested that USP10 played a crucial role in GC cell migration and invasion, which was mediated by the EMT-related pathway.

In addition, we also found that USP10 suppressed EMT through upregulating TNFRSF10B expression, that is, upregulated USP10 increased the expression of TNFRSF10B, then reduced Twist1 expression, resulting in upregulating E-cadherin expression, which was reversed by knock-down of TNFRSF10B, and downregulated TNFRSF10B facilitated the migration of GC cells.

Kaplan-Meier survival analysis showed that TNFRSF10B mRNA could predict the prognosis of GC patients in part of cohorts. However, the TNFRSF10B protein was not associated with prognosis in our GC cohort. As we know, survival time is the outcome of multiple factors interaction, EMT pathway is one of the variables that affect the prognosis [37]. After sub-classification, we found that TNFRSF10B protein was lowly expressed not only in GC with distant metastasis but also in the diffuse type and poorly cohesive carcinoma of GC. Therefore, we believed that TNFRSF10B could be used as a biomarker to identify patients with less aggressive GC. What`s more, hereditary diffuse GC (HDGC) is an inherited form of the diffuse type of GC and a highly invasive type of tumor [38], and inactivating mutations of the CDH1 gene encoding E-cadherin have been identified in 30–50% of patients with HDGC [38–40]. These results also support the correlation between TNFRSF10B and E-cadherin mediated cell adhesion.

Several limitations existed in the present study. First, we found that the effect of USP10 on cell migration was reversed when the concentration of si-USP10 was shifted from a low dose to a high dose. However, we solely identified the de-ubiquitination targets of USP10 at a low dose rather than a high dose. Hence, further multi-omics detections are needed to elucidate the molecular mechanism of high-dose USP10 in regulating GC metastasis. Second, as analyzed above, we only demonstrated the Twist 1 and E-cadherin could be regulated by TNFRSF10B. However, whether the regulatory effect of TNFRSF10B on Twist1 is direct or indirect has not been elucidated. Therefore, further validation experiments need to be conducted.

## Conclusion

Our study established a high-throughput strategy for screening de-ubiquitination targets for USP10 in GC metastasis, and found that USP10 inhibited the migration and invasion of GC cells through suppressing EMT due to de-ubiquitination of TNFRSF10B. These findings demonstrate a novel regulation mode of EMT and may improve understanding of ubiquitin regulatory networks in GC metastasis. Inhibiting the ubiquitination of TNFRSF10B may be considered as a potential therapeutic strategy for GC metastasis.

### Electronic supplementary material

Below is the link to the electronic supplementary material.

Supplementary Material 1

Supplementary Material 2

Supplementary Material 3

Supplementary Material 4

Supplementary Material 5

## Data Availability

The raw sequence data have been deposited in the Sequence Read Archive (SRA) in the National Center for Biotechnology Information (Maryland, USA) under the BioProject ID PRJNA941161. The datasets used in this study are available from the corresponding author upon reasonable request.

## Abbreviations

AGC: Automatic gain control
CDS: Coding sequence
CHX: Cycloheximide
CSTF2: Cleavage stimulation factor subunit 2
Co-IP: Co-immunoprecipitation
DR5: Death receptor 5
EDTA: Ethylenediaminetetraacetic acid
EMT: Epithelial-to-mesenchymal transition
EMT-TFs: EMT transcription factors
EV: Empty vector sample
FDR: False discovery rate
GAC: Gastric adenocarcinoma
GC: Gastric cancer
GFP: Green fluorescent protein
GPS1 G: Protein pathway suppressor 1
HCD: High-energy collision dissociation
H&E: Hematoxylin and eosin
IHC: Immunohistochemistry
IS: Intensity score
ISS: Immuno-staining score
MED16: Mediator complex subunit 16
MOI: Multiplicity of infection
NC: Normal control
OE: Overexpression sample
PS: Percentage score
QC: Quality control
RNF185: Ring finger protein 185
RPS27: Ribosomal protein S27
SLC2A3: Solute carrier family 2 member 3
TNFRSF10B: TNF receptor superfamily member 10b
TPD52: Tumor protein D52
USP10: Ubiquitin-specific protease 10
WB: Western blot
ZEB1: Zinc finger E-box binding homeobox 1
ZEB2: Zinc finger E-box binding homeobox 2
