# Supplementary material for: System-wide identification of novel de-ubiquitination targets for USP10 in gastric cancer metastasis through multi-omics screening
Source: BMC Cancer. 2024 Jun 27;24:773. doi: 10.1186/s12885-024-12549-3 (PMC11209979; doi:10.1186/s12885-024-12549-3)
Supplement: Supplementary file 1 — Supplementary Material 1 [file 12885_2024_12549_MOESM1_ESM.docx]

Supplementary Figures


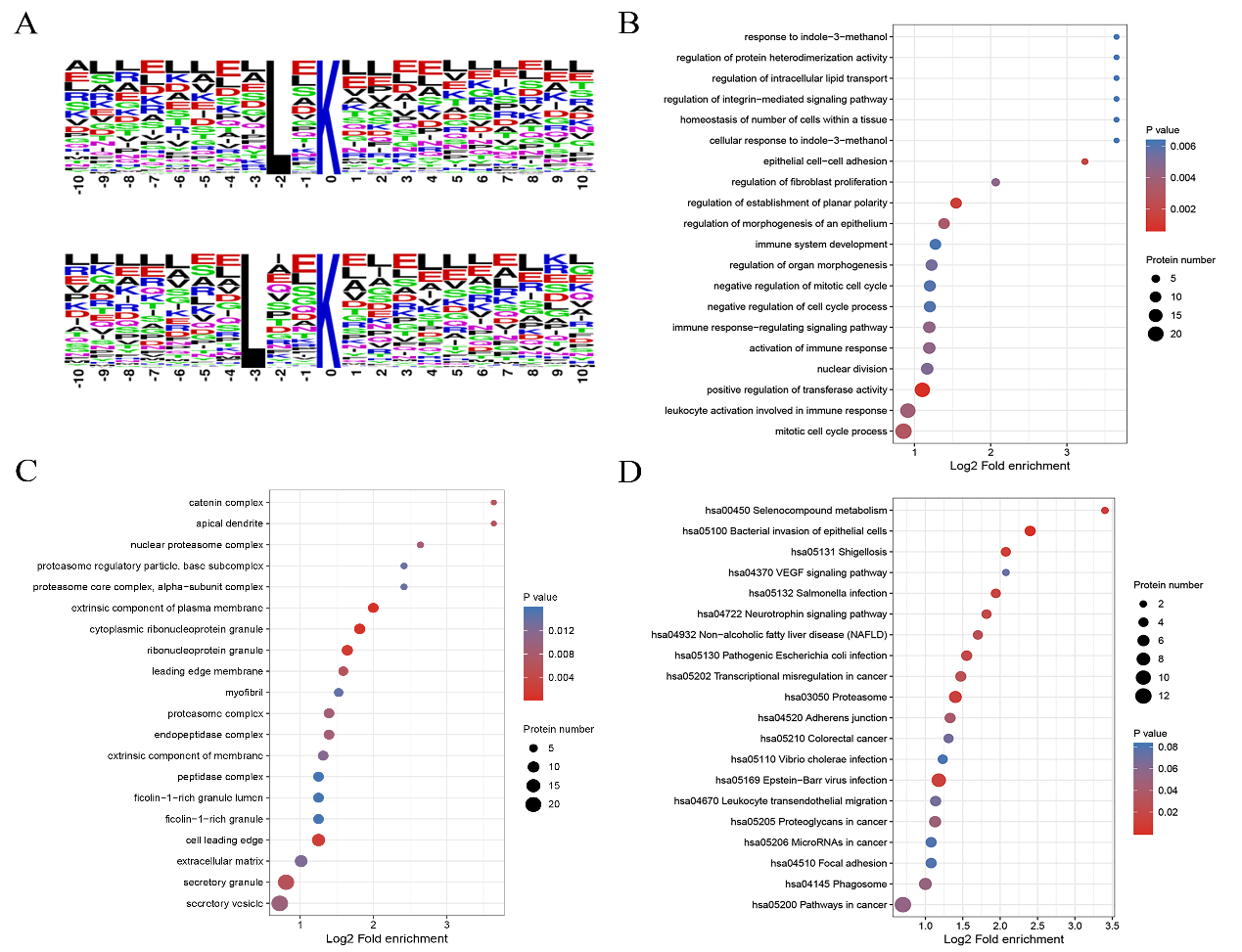


**Figure S1** **The proteomics analysis and ubiquitinated proteomics analysis revealed modified sequence, EMT-related GO functions and pathways.** A) Motif Logo for modified sequence. B) GO Biological Process enrichment analysis of USP10 sites in ubiquitinated proteomics analysis. C) GO Cellular Component enrichment analysis of USP10 sites in ubiquitinated proteomics analysis. D) KEGG Pathway enrichment analysis of USP10 downregulating ubiquitinated sites in ubiquitinated proteomics analysis.


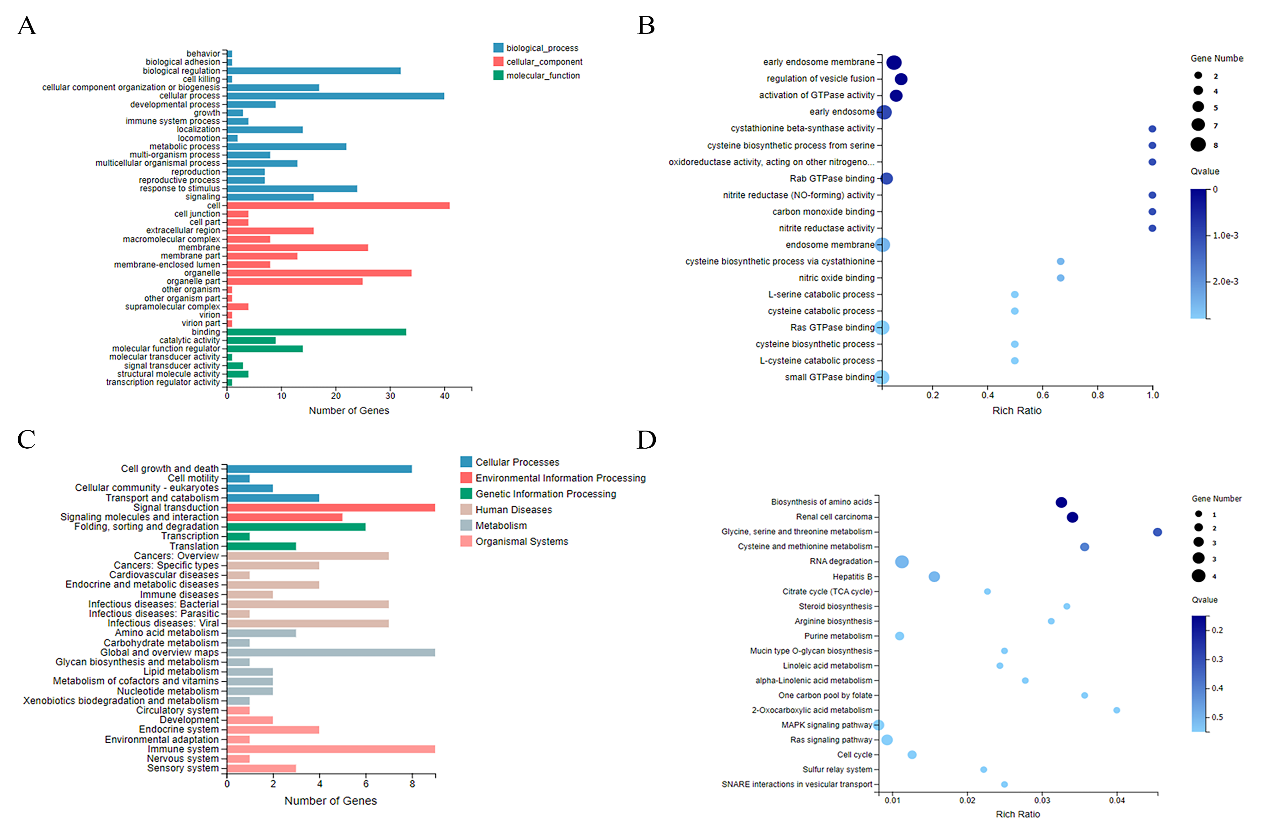


**Figure S2** **The transcriptomic analysis.** A) GO classification. B) GO enrichment analysis. C) KEGG Pathway classification analysis. D) KEGG Pathway enrichment analysis.


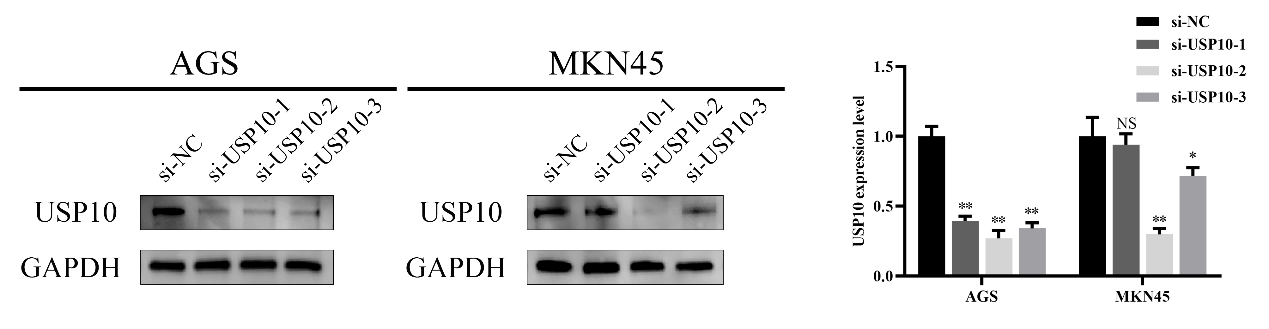


**Figure S3** **The knockdown efficiency of si-USP10 was confirmed.** The protein level of USP10 was measured when si-USP10-1, si-USP10-2, or si-USP10-3 was transfected into AGS cells or MKN45 cells, WB analysis (left) and densitometry analysis (right). n=6 per group, **p* < 0.05, ***p* < 0.01.


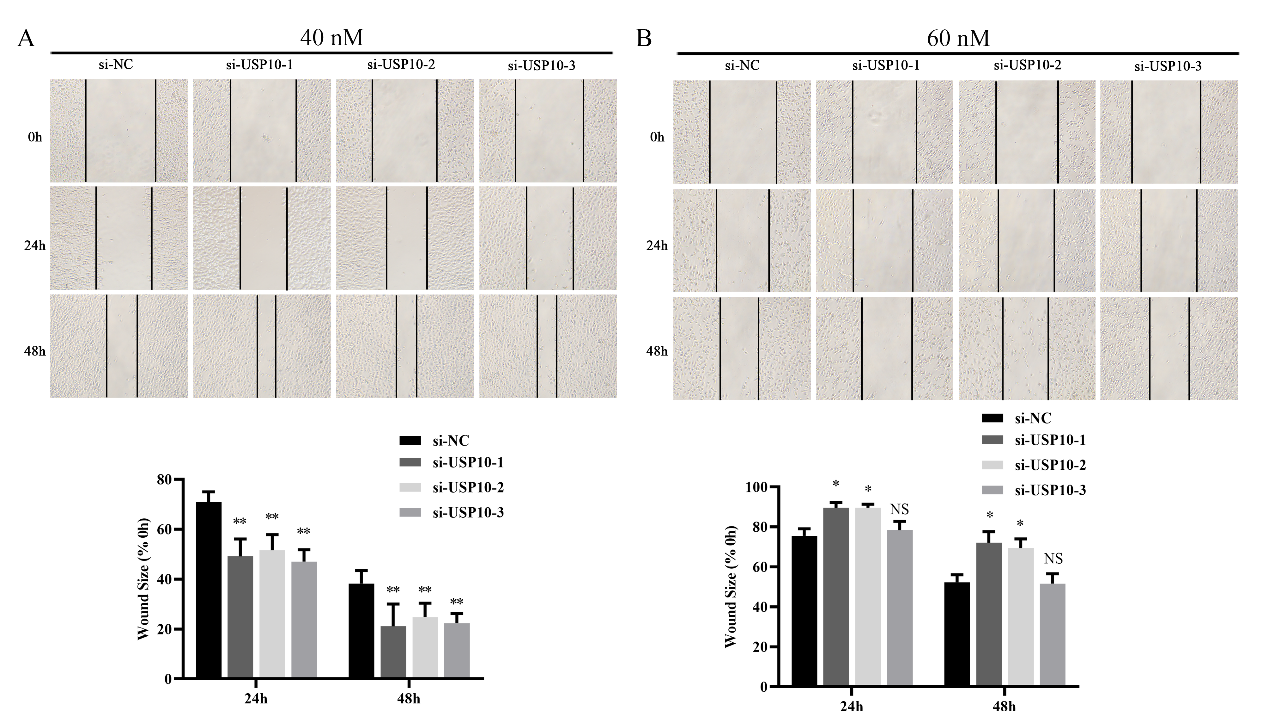


**Figure S4** **The effect of si-USP10 concentration on the migration and growth of gastric cancer cells.** A) Wound-healing assay (up) and quantitative analysis (down) in AGS cells transfected with si-USP10-1, si-USP10-2, or si-USP10-3 at 40 nM. B) Wound-healing assay (up) and quantitative analysis (down) in AGS cells transfected with si-USP10-1, si-USP10-2, or si-USP10-3 at 60 nM. **p* < 0.05, ***p* < 0.01.


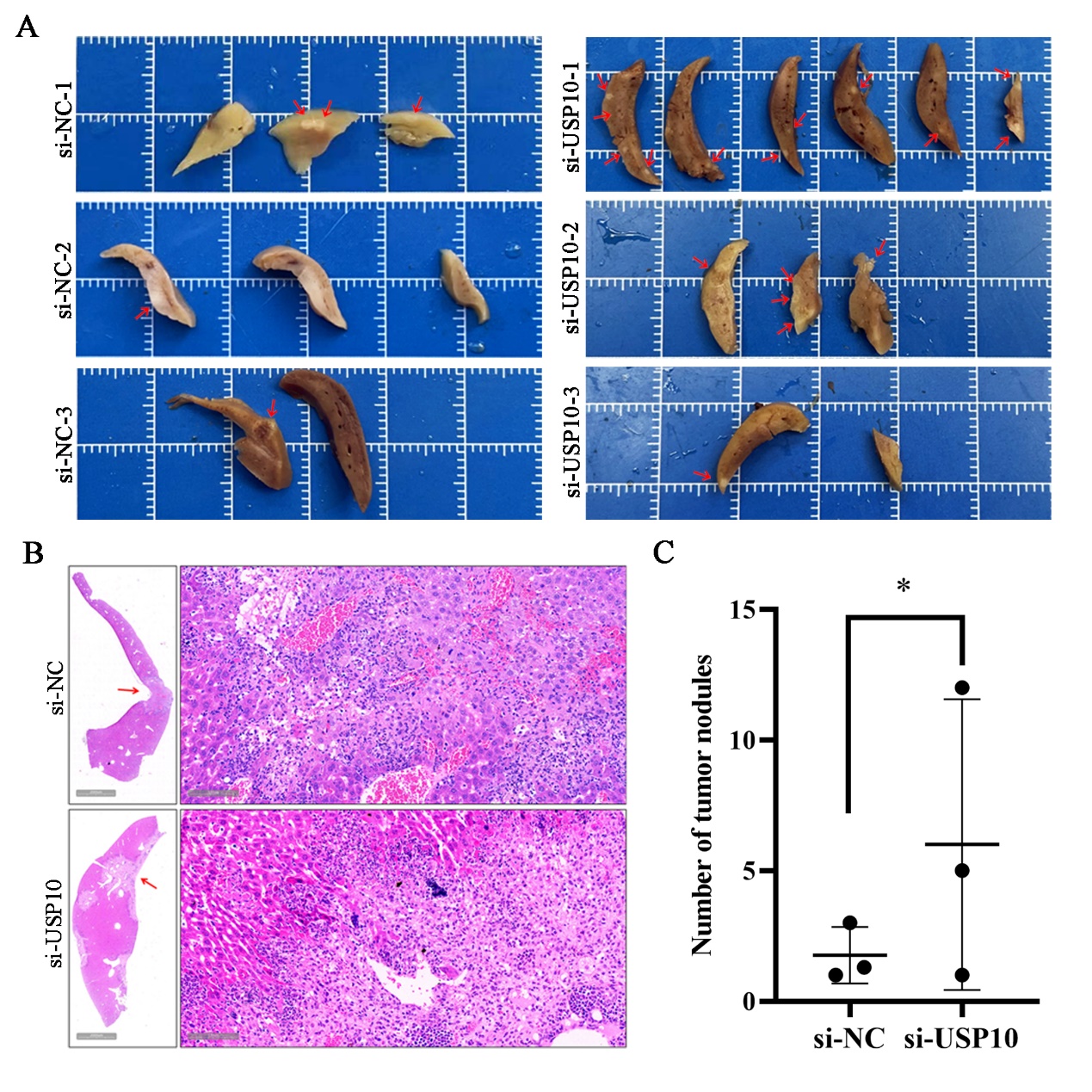


**Figure S5** **Downregulated USP10 promotes GC liver metastasis *in vivo*.** A) Liver tissues were shown when mice were euthanized after AGS cells transfected with si-USP10 were injected intravenously for 5 weeks, red arrow: metastatic nodules (n=5 per group, tumor formation rate=60%). B) The H&E staining in liver tissue sections. C) The numbers of tumor nodules were counted and statistical analysis was performed. **p* < 0.05.


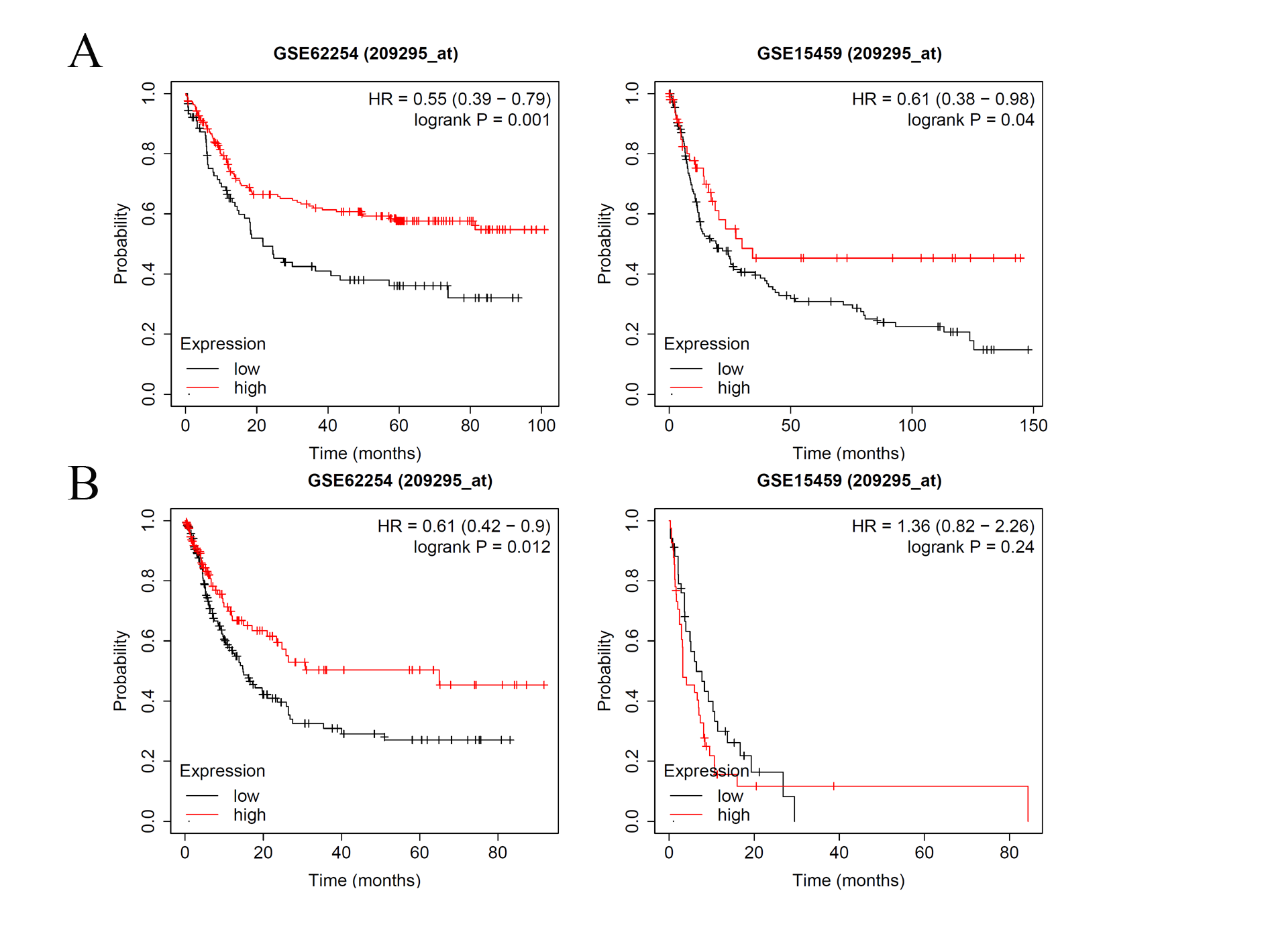
**Figure S6 The first progression survival and post progression survival analysis of TNFRSF10B in two GEO datasets of GAC.** A) The first progression survival analysis of TNFRSF10B mRNA using Kaplan-Meier method in GSE62254 and GSE15459. B) The post progression survival analysis of TNFRSF10B mRNA using Kaplan-Meier method in GSE62254 and GSE15459. GEO datasets were downloaded and analyzed using Kaplan-Meier plotter (https://kmplot.com/analysis).
