# Supplementary material for: System-wide identification of novel de-ubiquitination targets for USP10 in gastric cancer metastasis through multi-omics screening
Source: BMC Cancer. 2024 Jun 27;24:773. doi: 10.1186/s12885-024-12549-3 (PMC11209979; doi:10.1186/s12885-024-12549-3)
Supplement: Supplementary file 2 — Supplementary Material 2 [file 12885_2024_12549_MOESM2_ESM.docx]

**Supplementary Tables S1-S2**

Table S1 The siRNA sequences of the USP10 and TNFRSF10B

| Gene | siRNA ID |  | Sequence (5’-3’) |
| --- | --- | --- | --- |
| USP10 | si-USP10-1 | sense | UAUGUGGAAACUAAGUAUUTT |
|  |  | antisense | AAUACUUAGUUUCCACAUATT |
| USP10 | si-USP10-2 | sense | UGUGGAUAAACUACCUGAUTT |
|  |  | antisense | AUCAGGUAGUUUAUCCACATT |
| USP10 | si-USP10-3 | sense | CUCUCUUUAGUGGCUCUUUTT |
|  |  | antisense | AAAGAGCCACUAAAGAGAGTT |
| TNFRSF10B | si-TNFRSF10B-1 | sense | GCAAAUAUGGACAGGACUATT |
|  |  | antisense | UAGUCCUGUCCAUAUUUGCTT |
| TNFRSF10B | si-TNFRSF10B-2 | sense | CCCUGGAGUGACAUCGAAUTT |
|  |  | antisense | AUUCGAUGUCACUCCAGGGTT |
| TNFRSF10B | si-TNFRSF10B-3 | sense | GGGACACCUUGUACACGAUTT |
|  |  | antisense | AUCGUGUACAAGGUGUCCCTT |

Table S2 The primers for qRT-PCR

| Gene | primer | Sequence (5’-3’) |
| --- | --- | --- |
| USP10 | Forward | ATTGAGTTTGGTGTCGATGAAGT |
|  | Reverse | GGAGCCATAGCTTGCTTCTTTAG |
| TNFRSF10B | Forward | GCCCCACAACAAAAGAGGTC |
|  | Reverse | AGGTCATTCCAGTGAGTGCTA |
| MED16 | Forward | GCTGCACAATGGTGTGAAACT |
|  | Reverse | AGAACTTGACTCGGGAGAACTT |
| Twist1 | Forward | GTCCGCAGTCTTACGAGGAG |
|  | Reverse | GCTTGAGGGTCTGAATCTTGCT |
| GAPDH | Forward | GGAGCGAGATCCCTCCAAAAT |
|  | Reverse | GGCTGTTGTCATACTTCTCATGG |
