# Supplementary material for: System-wide identification of novel de-ubiquitination targets for USP10 in gastric cancer metastasis through multi-omics screening
Source: BMC Cancer. 2024 Jun 27;24:773. doi: 10.1186/s12885-024-12549-3 (PMC11209979; doi:10.1186/s12885-024-12549-3)

Figure 2-C

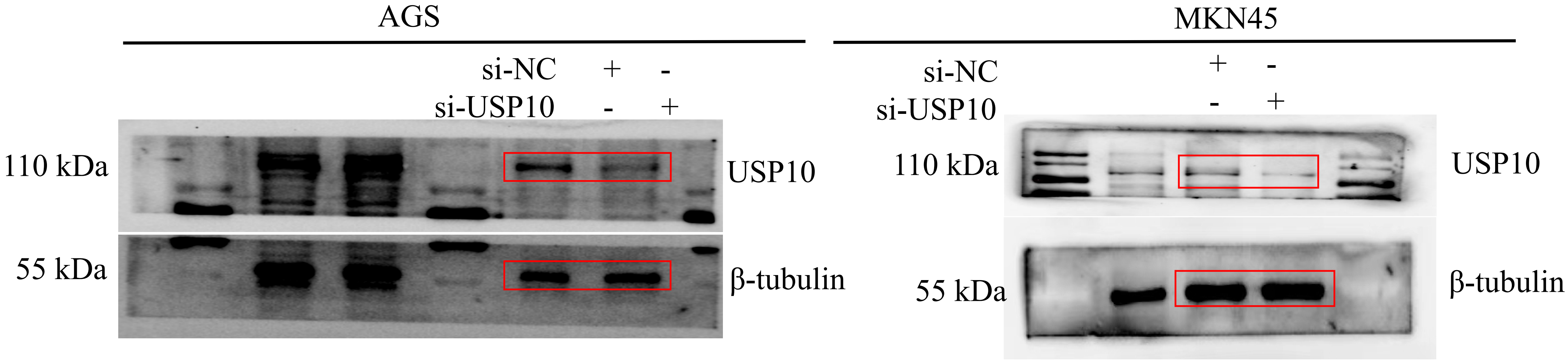
AGS
si-NC + -
si-USP10 - +
110 kDa
USP10
55 kDa
β-tubulin
MKN45
si-NC + -
si-USP10 - +
110 kDa
USP10
55 kDa
β-tubulin

Figure 2-F

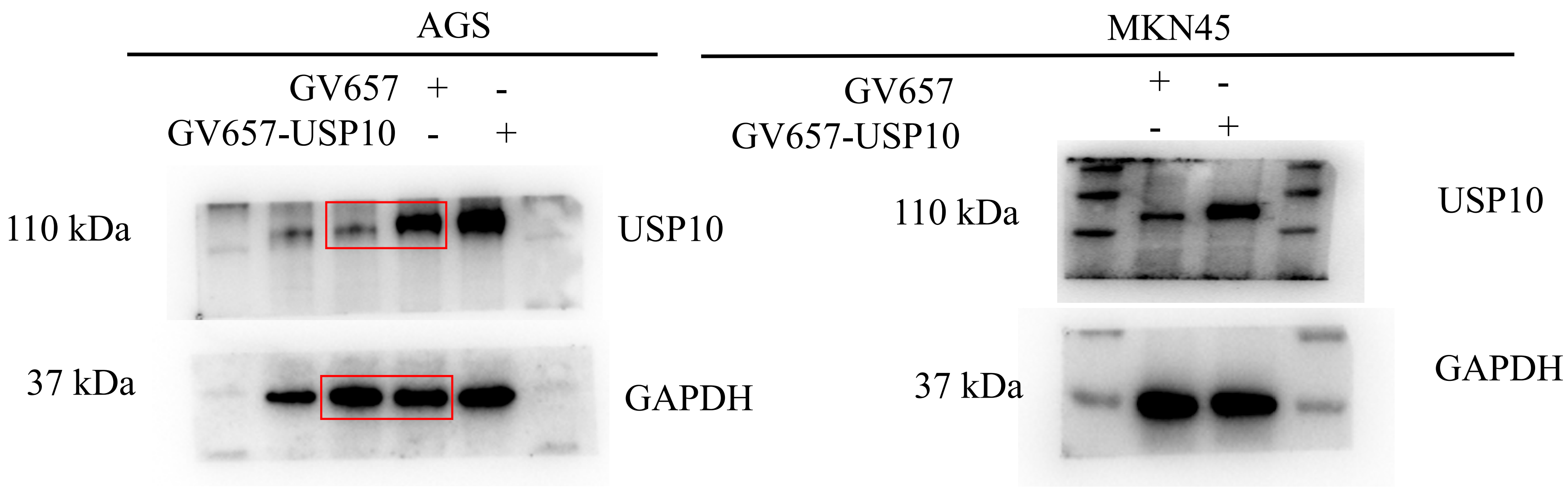

AGS
GV657 + -
GV657-USP10 - +
110 kDa
USP10
37 kDa
GAPDH
MKN45
GV657 + -
GV657-USP10 - +
110 kDa
USP10
37 kDa
GAPDH

Figure 3-A

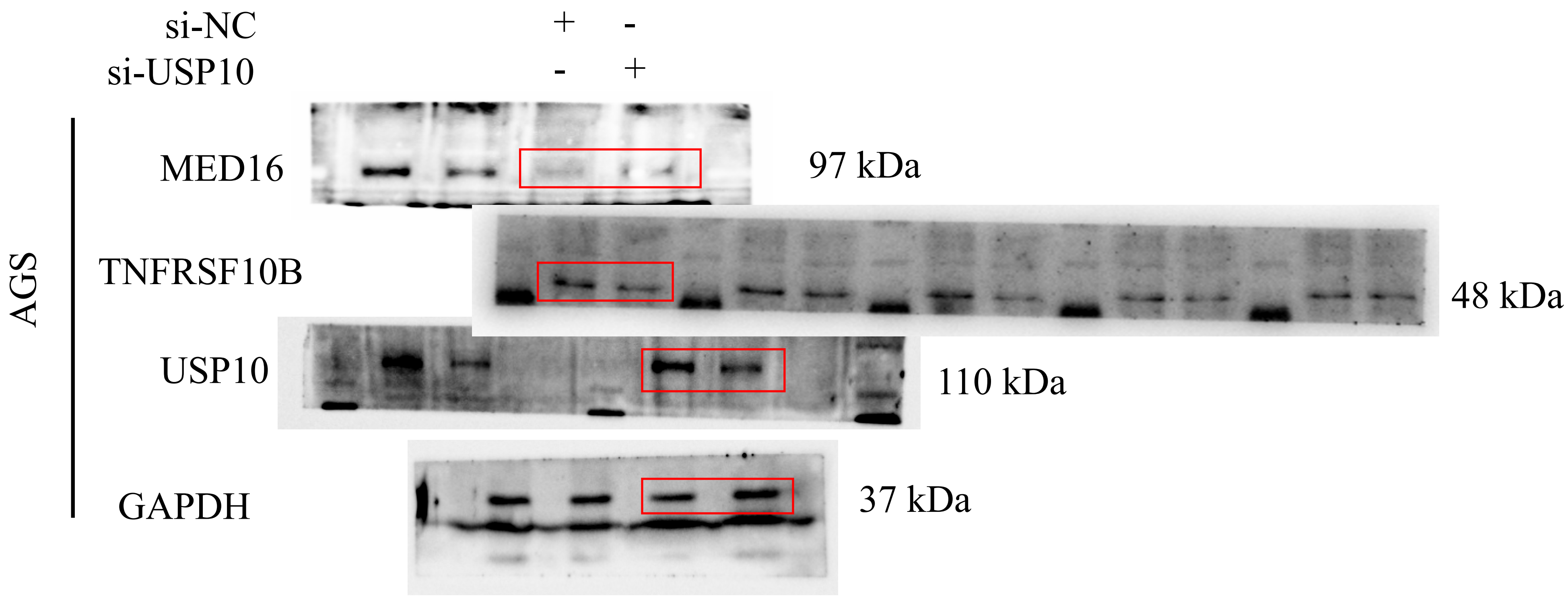
si-NC + -
si-USP10 - +
AGS
MED16
97 kDa
TNFRSF10B
48 kDa
USP10
110 kDa
GAPDH
37 kDa

Figure 3-B

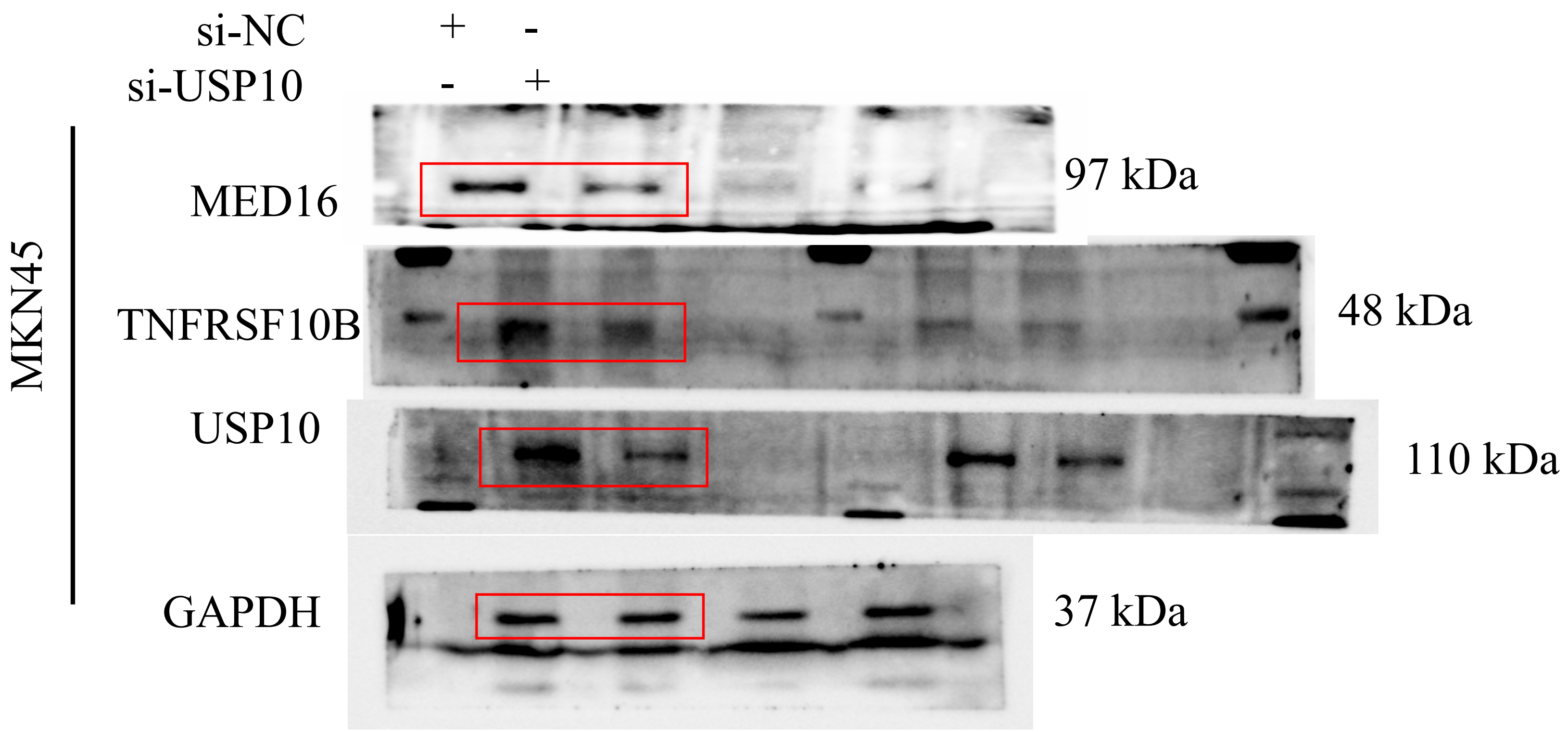
si-NC + -
si-USP10 - +
MKN45
MED16
97 kDa
TNFRSF10B
48 kDa
USP10
110 kDa
GAPDH
37 kDa

Figure 3-E

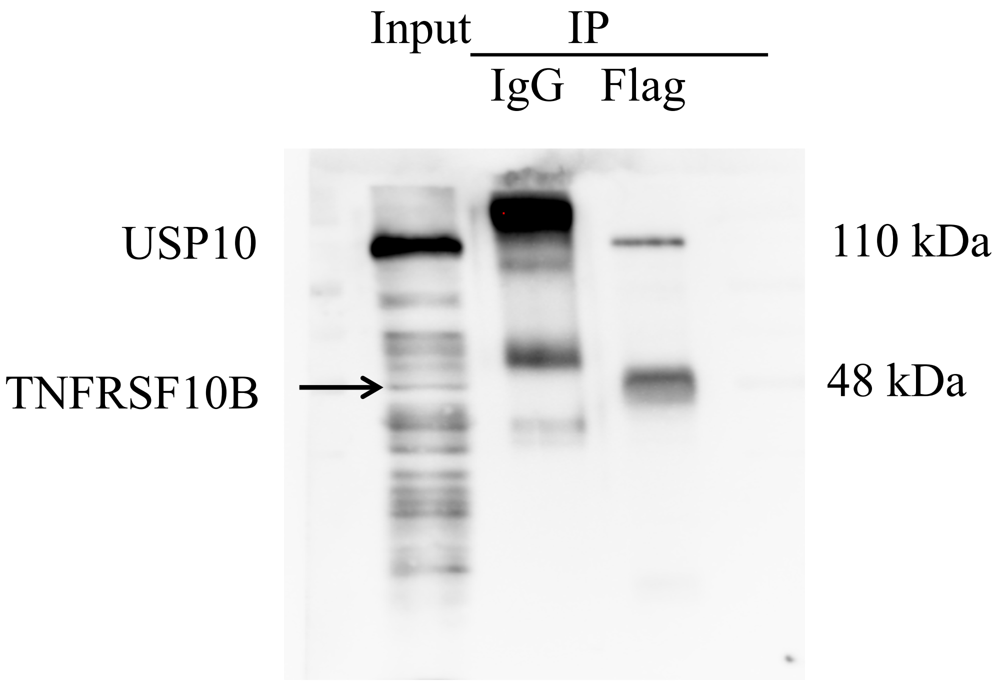

Figure 3-F

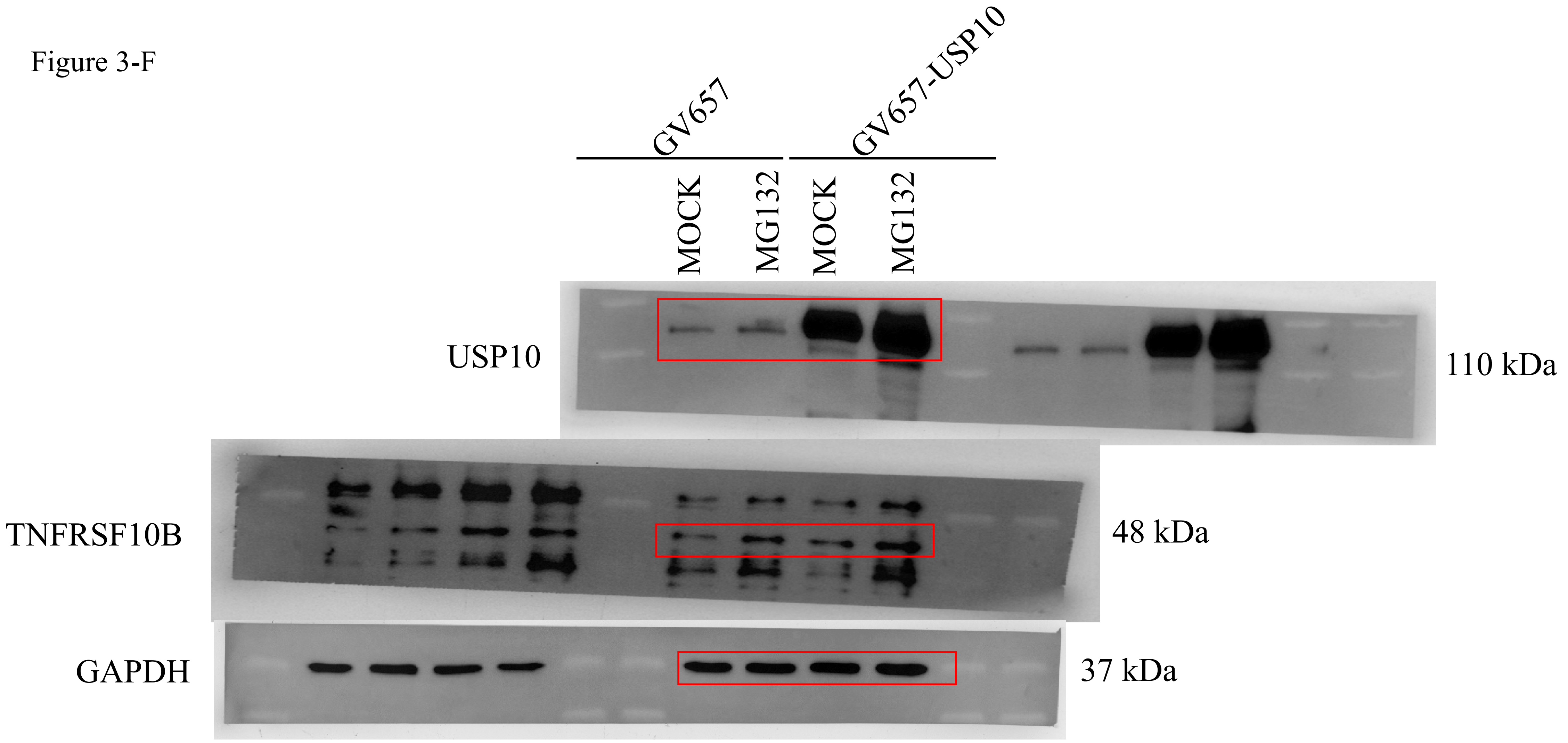
GV657
GV657-USP10
MOCK
MG132
MOCK
MG132
USP10
110 kDa
TNFRSF10B
48 kDa
GAPDH
37 kDa

Figure 3-G

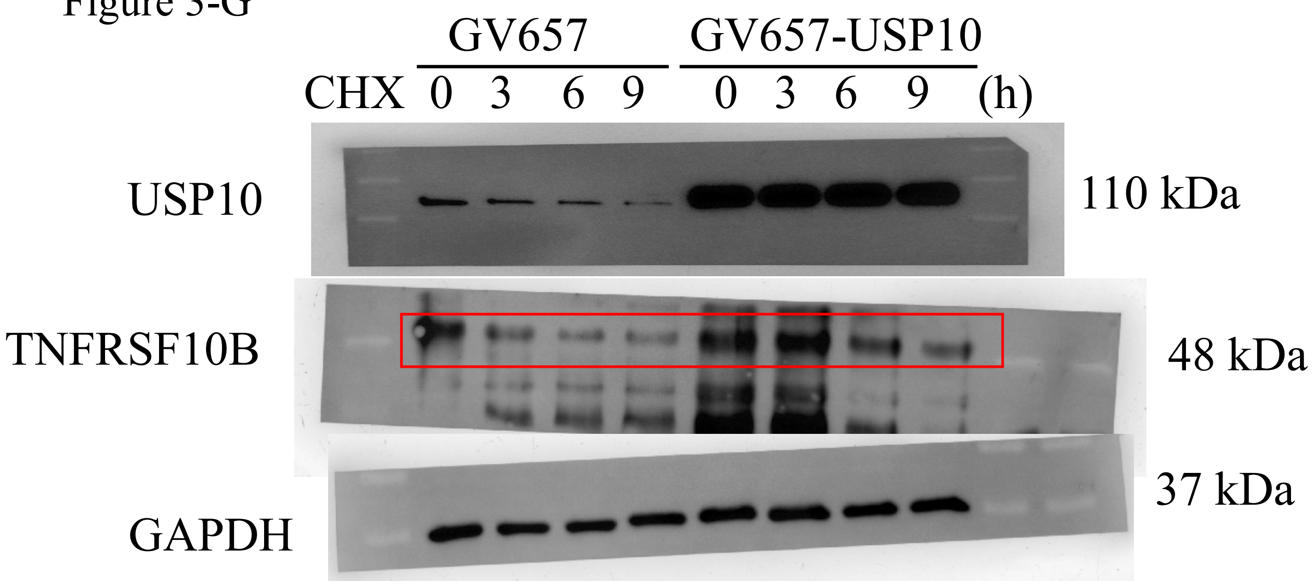
GV657
GV657-USP10
CHX 0 3 6 9 0 3 6 9 (h)
USP10
110 kDa
TNFRSF10B
48 kDa
37 kDa
GAPDH

Figure 4-C

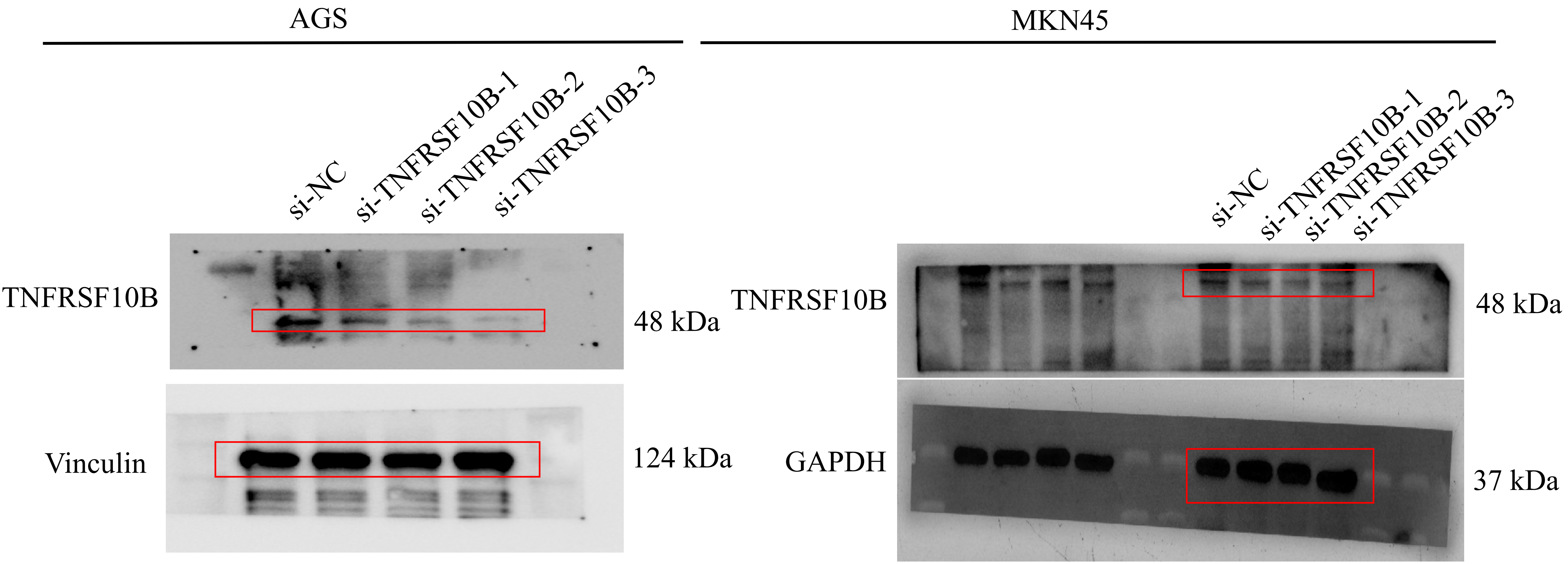
AGS
MKN45
si-NC
si-TNFRSF10B-1
si-TNFRSF10B-2
si-TNFRSF10B-3
TNFRSF10B
48 kDa
Vinculin
124 kDa
GAPDH
37 kDa

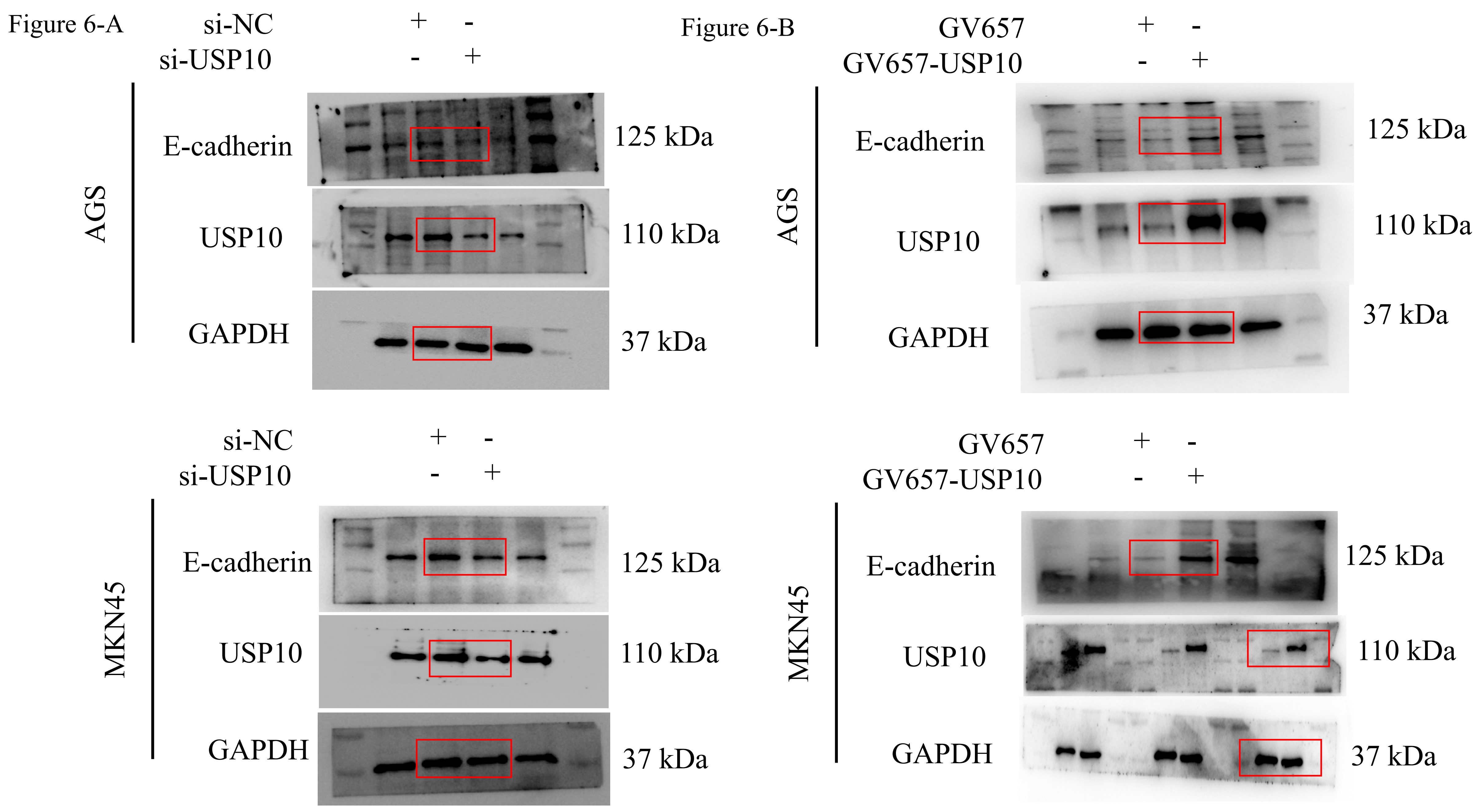
Figure 6-A
si-NC + -
si-USP10 - +
AGS
E-cadherin
125 kDa
USP10
110 kDa
GAPDH
37 kDa
MKN45
si-NC + -
si-USP10 - +
E-cadherin
125 kDa
USP10
110 kDa
GAPDH
37 kDa
Figure 6-B
GV657 + -
GV657-USP10 - +
AGS
E-cadherin
125 kDa
USP10
110 kDa
GAPDH
37 kDa
MKN45
GV657 + -
GV657-USP10 - +
E-cadherin
125 kDa
USP10
110 kDa
GAPDH
37 kDa

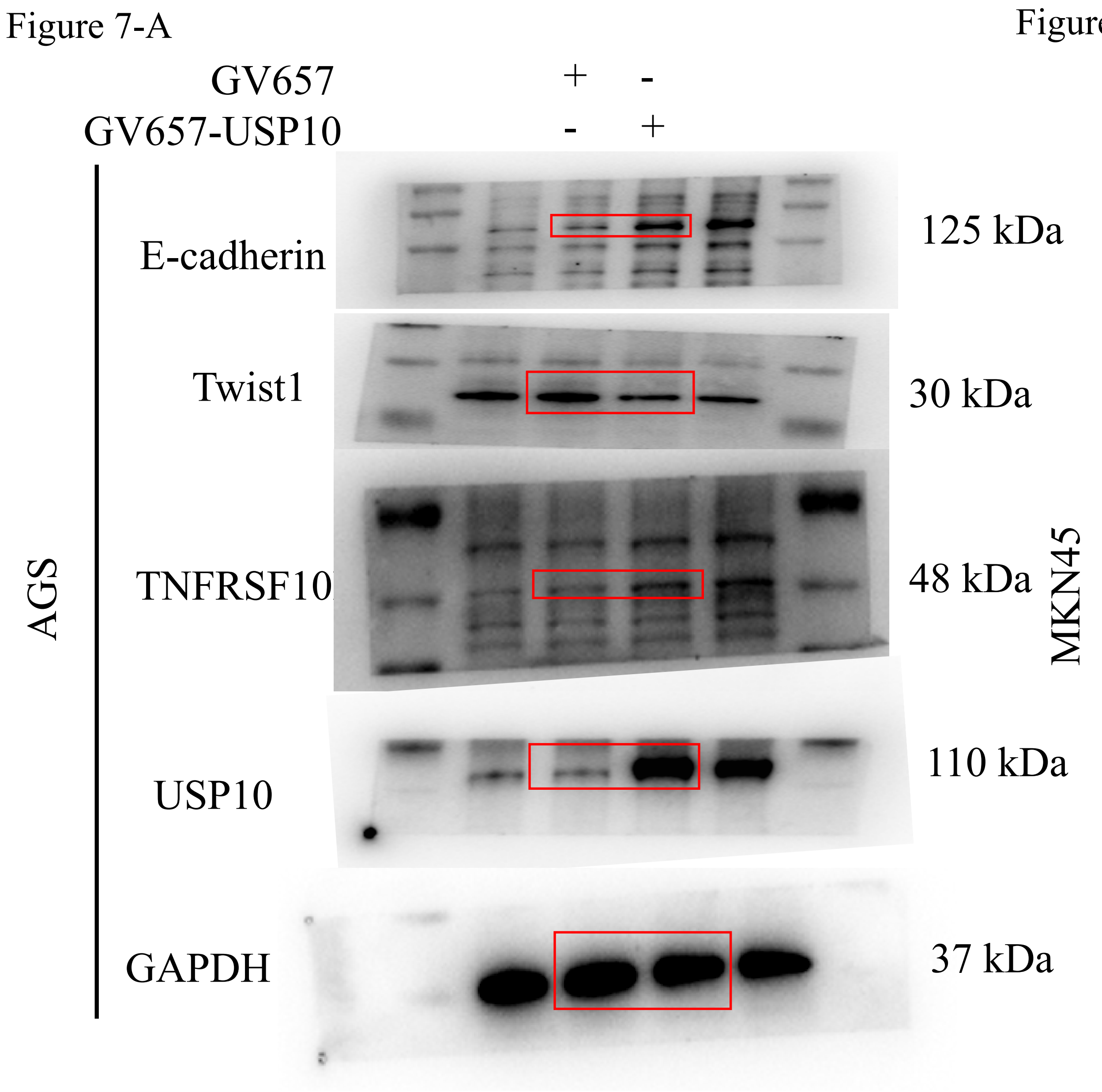
Figure 7-A
GV657 + -
GV657-USP10 - +
AGS
E-cadherin
125 kDa
Twist1
30 kDa
TNFRSF10
48 kDa
USP10
110 kDa
GAPDH
37 kDa

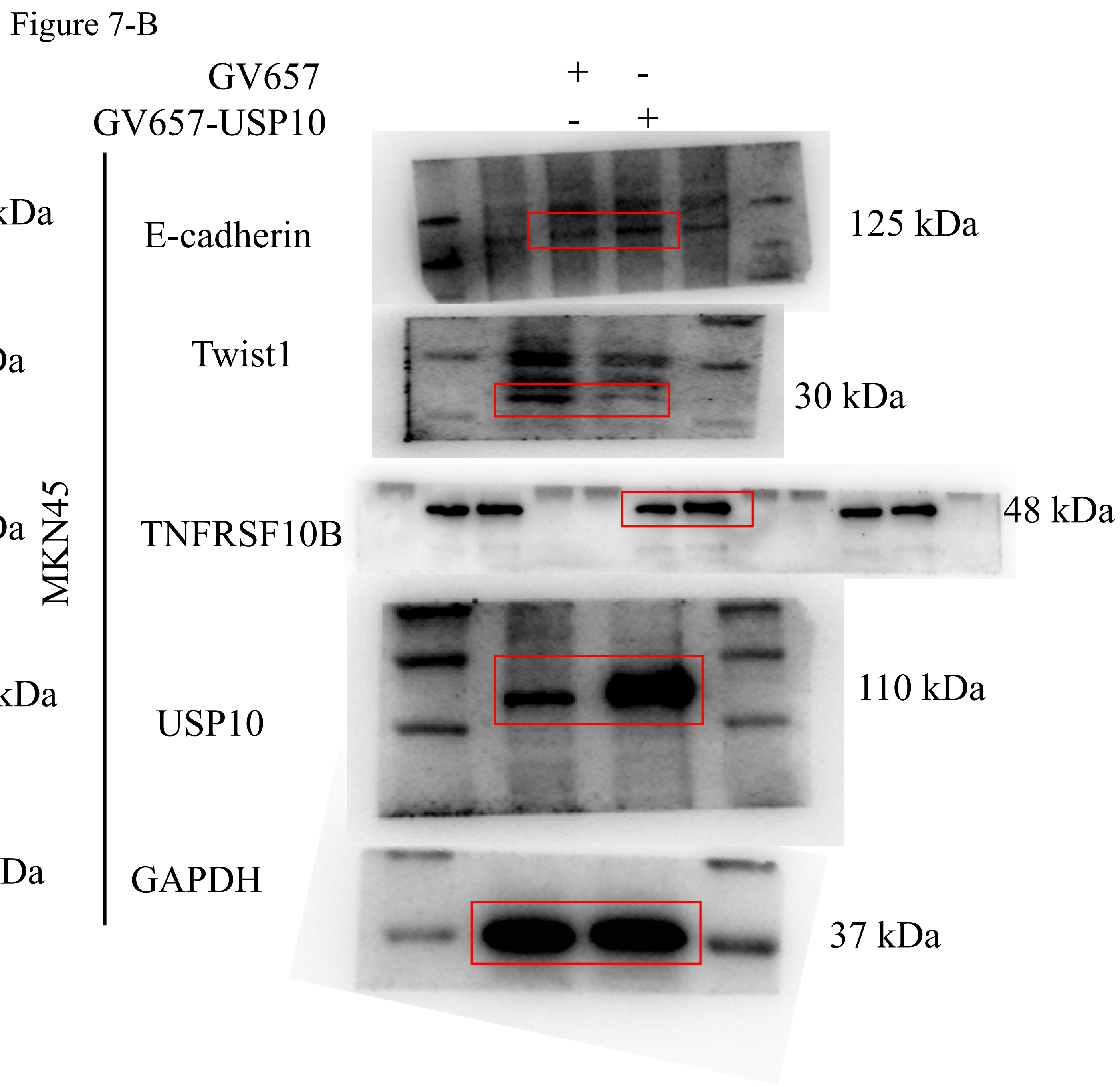
Figure 7-B
GV657 + -
GV657-USP10 - +
MKN45
E-cadherin
125 kDa
Twist1
30 kDa
TNFRSF10B
48 kDa
USP10
110 kDa
GAPDH
37 kDa

Figure 7-E

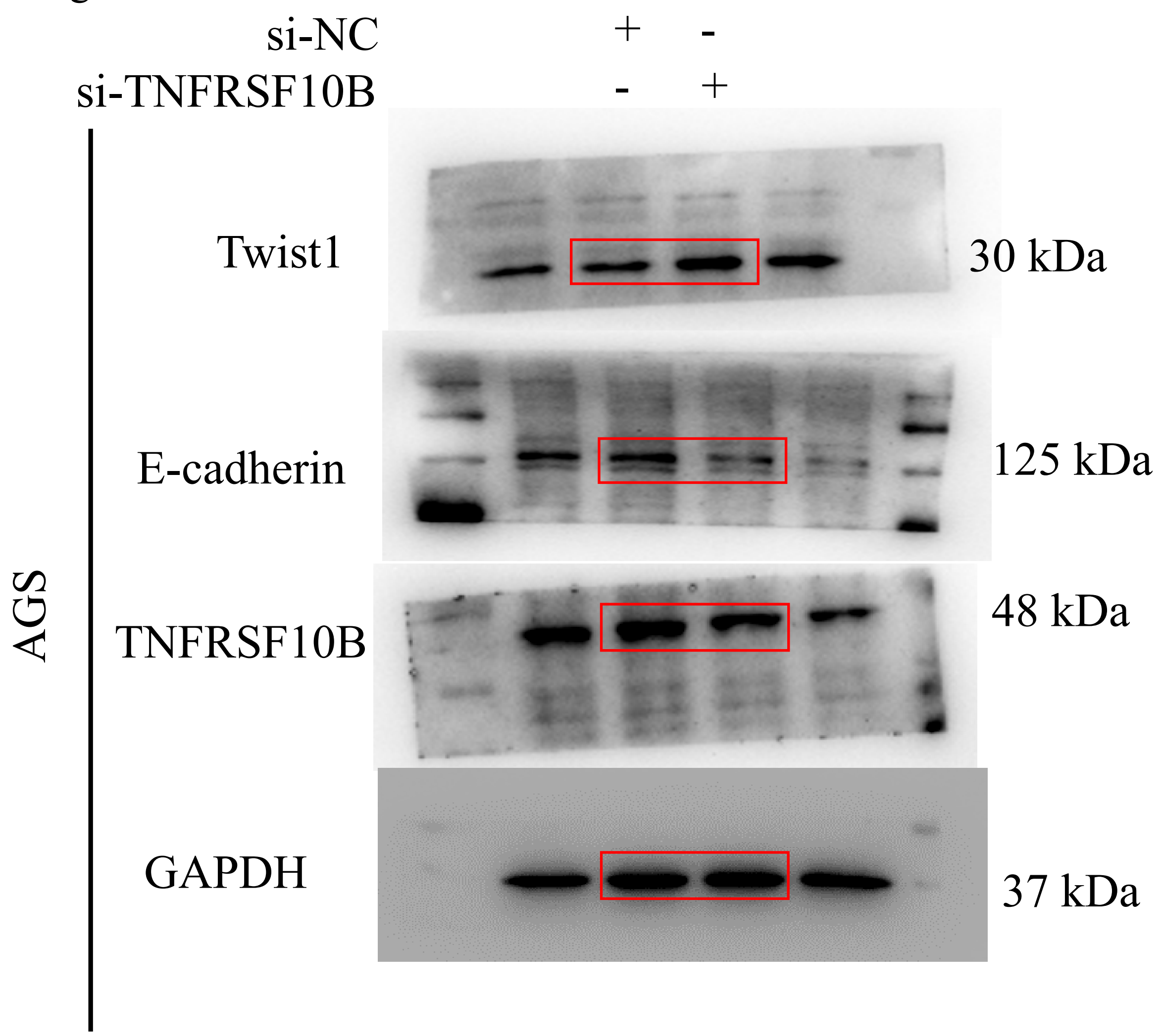
si-NC + -
si-TNFRSF10B - +
AGS
Twist1
30 kDa
E-cadherin
125 kDa
TNFRSF10B
48 kDa
GAPDH
37 kDa

Figure 7-F

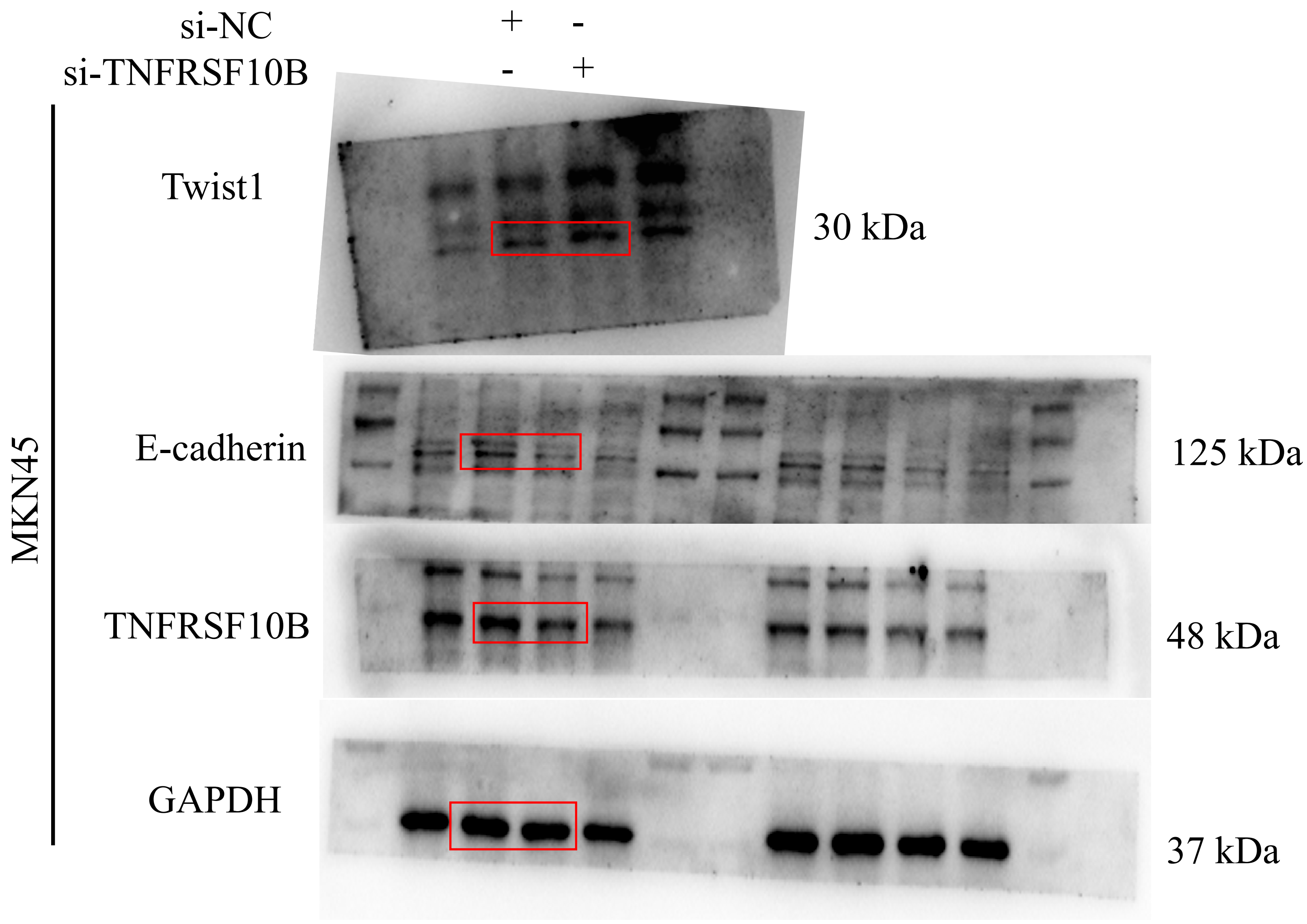
si-NC
+
-
si-TNFRSF10B
-
+
MKN45
Twist1
30 kDa
E-cadherin
125 kDa
TNFRSF10B
48 kDa
GAPDH
37 kDa

Figure 7-I

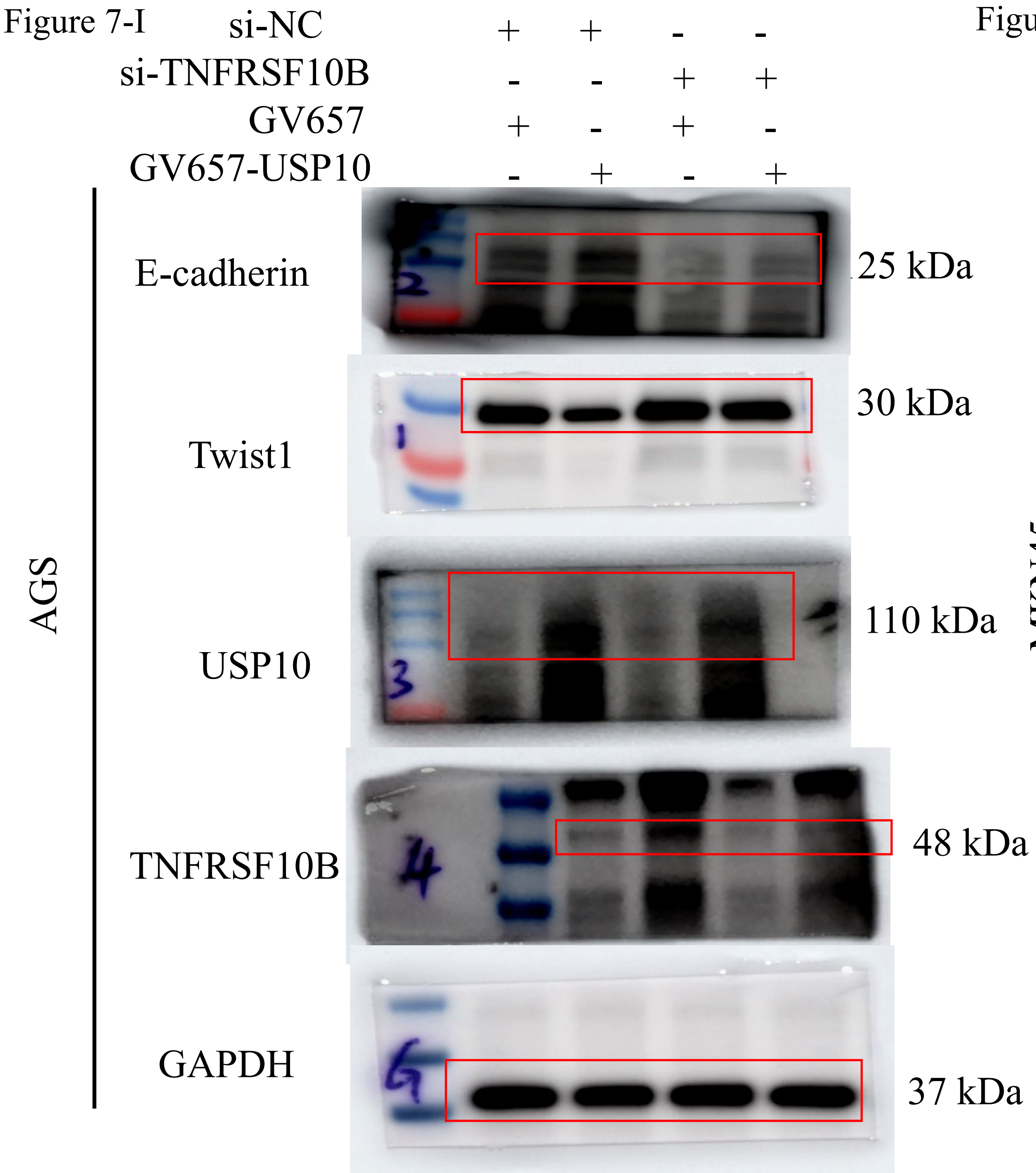

Figure 7-K

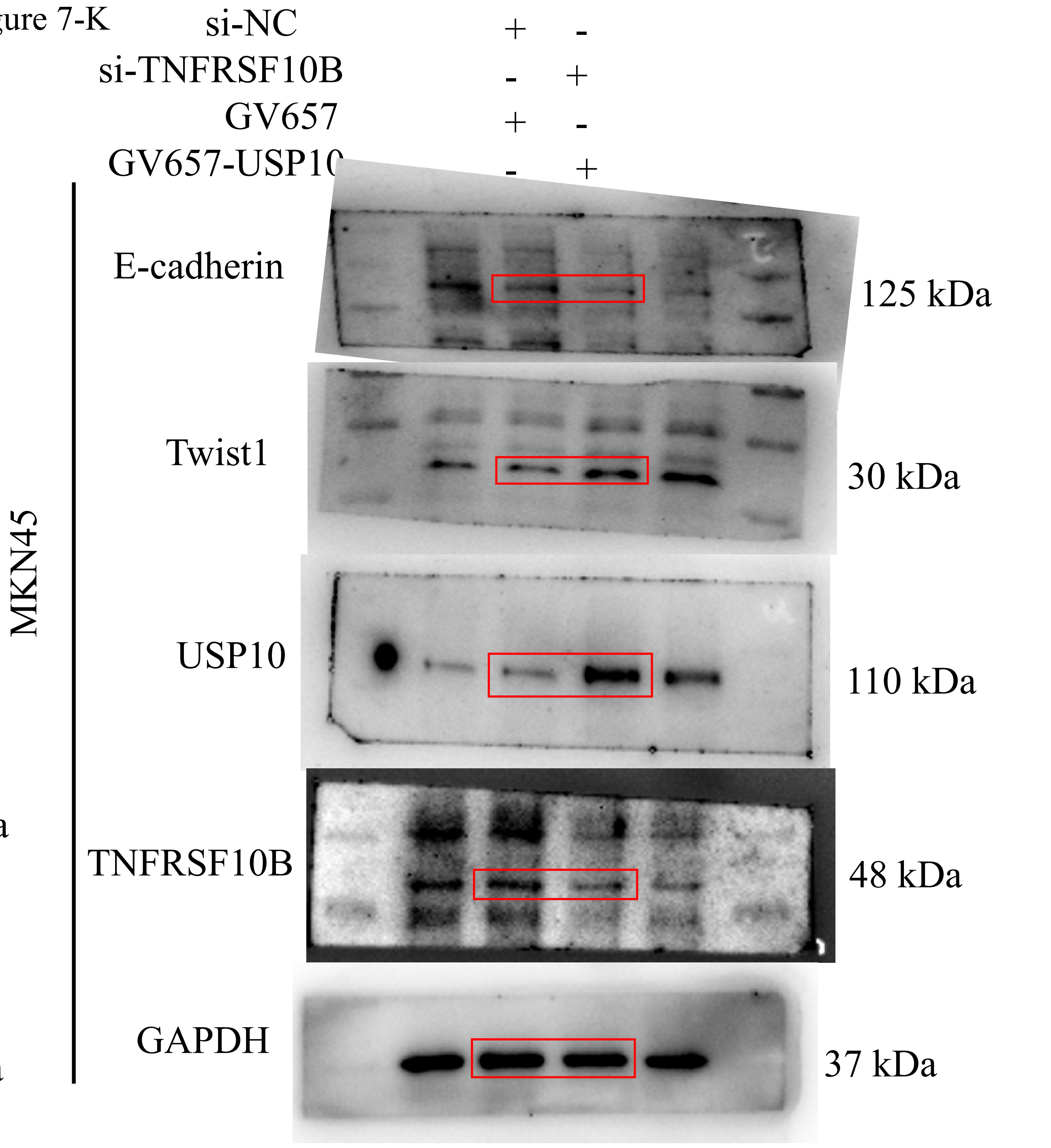

Supplementary Figure S3

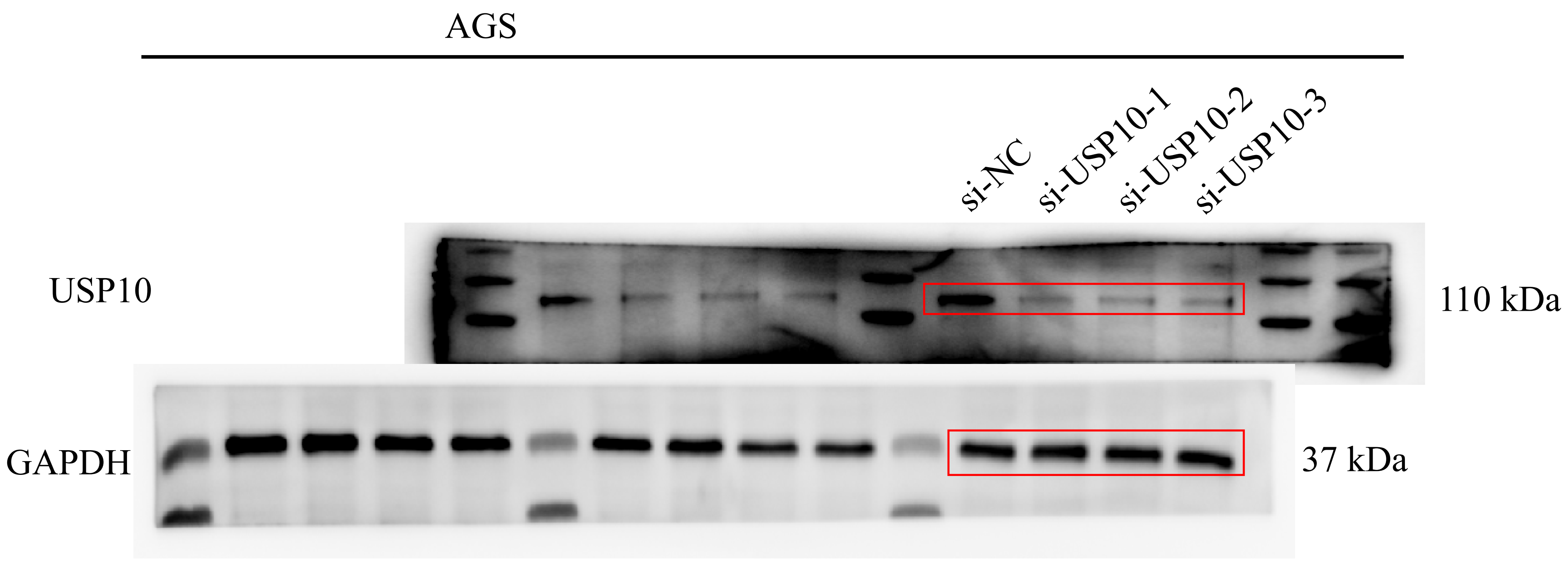

Supplementary Figure S3

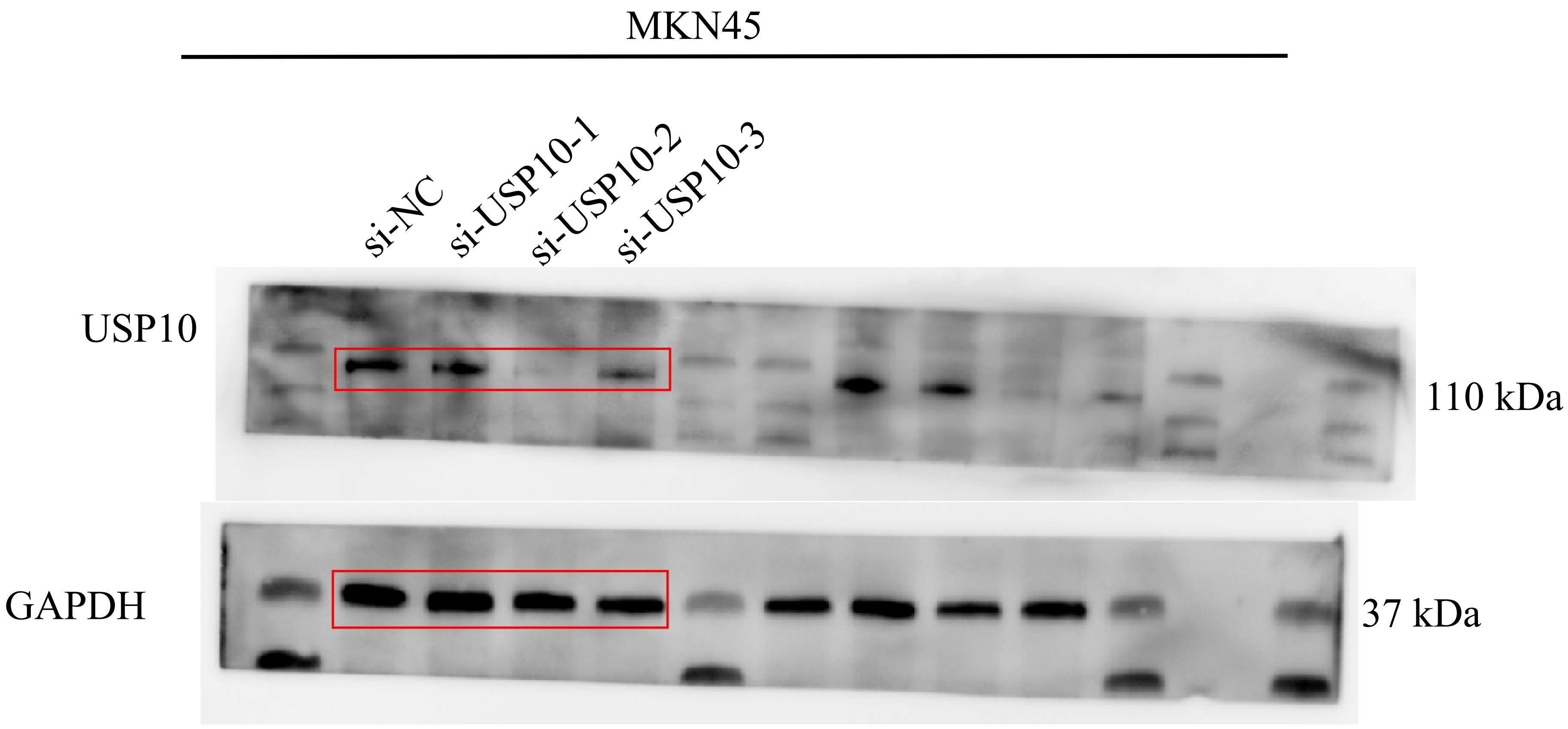

Supplement: Supplementary file 4 — Supplementary Material 4 [file 12885_2024_12549_MOESM4_ESM.pdf]
