# Supplementary material for: System-wide identification of novel de-ubiquitination targets for USP10 in gastric cancer metastasis through multi-omics screening
Source: BMC Cancer. 2024 Jun 27;24:773. doi: 10.1186/s12885-024-12549-3 (PMC11209979; doi:10.1186/s12885-024-12549-3)
Supplement: Supplementary file 5 — Supplementary Material 5 [file 12885_2024_12549_MOESM5_ESM.docx]

**Supplementary Materials and Methods**

**Reagents**

Proteasome inhibitor MG132 was purchased from MedChemExpress LLC (Shanghai, China). Protein synthesis inhibitor cycloheximide (CHX) was purchased from Sigma-Aldrich (St. Louis, MO, USA).

**Wound healing assay and transwell invasion assay**

AGS cells or MKN45 cells were seeded into 6-well plates (5×10^5^ cells/well). After transfection, when the cells confluence transcended 80%, the cell layer was wounded gently by a 0.2 mL micropipette tip across the diameter of the well. Cells were washed twice with 1×PBS, then cultured with 1% serum medium. Pictures were captured by the inverted microscope (Olympus Corporation, Japan) at 0 h, 24 h, and 48 h after the scratching.

The invasion of GC cell lines was measured by 24-well Transwell chambers (Jet Bio-Filtration, Guangzhou, China). A thin layer of Matrigel was added into the upper chambers. After transfection, cells were resuspended in 1% serum medium (1×10^5^ cells) and seeded into the upper chamber. Meanwhile, 600 μL 10% serum medium acted as the attractant and was supplemented into the lower chamber. After incubation of 24 h or 48 h at 37 °C, cells on the upper side were wiped with a cotton swab, and the cells on the lower side were stained with 2% crystal violet for 10 min. The pictures of stained cells in six random fields were captured by the inverted microscope (Olympus Corporation, Japan).

**Real-time quantitative RT-PCR (****qRT-PCR)**

Total RNA was extracted with RNAiso Plus (9109, TaKaRa, Shiga, Japan) and chloroform according to the manufacturer’s instructions. After measuring the concentration and purity of the extracted total RNA, 1 μg of total RNA was reverse-transcribed using the PrimerScript RT reagent Kit containing gDNA Eraser (RR820a, TaKaRa, Shiga, Japan) to remove genomic DNA. Then, the resulting cDNA was mixed with synthetic primers (Beijing Genomics Institute, Shenzhen, China) and SYBR Premix Ex Taq2 (RR047a, TaKaRa, Shiga, Japan) for qRT-PCR. The primer sequences are listed in Table S2. The thermocycling parameters were used as follows: 50 ℃ for 2 min, 95 ℃ for 10 min, followed by 45 cycles of 95 ℃ for 10 s, 60 ℃ for 10 s, and 72 ℃ for 15 s. The GAPDH was used as the endogenous control and the relative expression level of the target gene was calculated using the 2^–ΔΔCT^ equation.

**Protein extraction and Western blot (WB) analysis**

Total protein was extracted with RIPA lysis buffer (C1053, Applygen, Beijing, China) and protease inhibitor cocktail (G2006, Servicebio, Wuhan, China). Protein content was determined with the BCA kit (G2026, Servicebio, Wuhan, China). The whole cell lysates were resolved on 8-12% SDS-PAGE gels and the proteins were transferred onto polyvinylidene fluoride (PVDF) membranes (Millipore, Billerica, MA, USA) for 2 h. The membranes were put into 5% non-fat milk with PBS/0.1% Tween and blocked for 1 h, followed by incubation overnight at 4 ℃ with the primary antibody. Then, the membranes were incubated with the horseradish peroxidase (HRP) conjugated IgG antibody (1:3000 dilution, 7074P2 or 7076P2, Cell Signaling Technology, Danvers, MA, USA), or with an IRDye-labeled secondary antibody (diluted 1:10000, 926-32211, LI-COR, Lincoln, NE, USA) for 1 hour. GAPDH, β-tubulin, or Vinculin was served as a loading control. Images were acquired by the ChemiDoc Touch System (Bio-Rad Laboratories, Hercules, CA, USA), or the Odyssey Western Blot Analysis system (LI-COR, Lincoln, NE, USA). The band intensity was quantified by densitometry analysis using Quantity One v4.6.2 software (Bio-Rad Laboratories, Hercules, CA, USA), and normalized to the GAPDH, β-tubulin, or Vinculin loading control. The blots were cut prior to hybridization with antibodies, so original images of full-length blots cannot be provided, but the membrane edges of the images of the original blots in Supplementary Material 5 were visible.

**RNA sequencing and expression analysis**

After stable overexpression of USP10 in AGS cells, total RNA was extracted with RNAiso Plus (9109, TaKaRa, Shiga, Japan) and chloroform according to the manufacturer’s instructions. After measuring the concentration and purity of the extracted total RNA, the RNA sequencing was conducted by the Beijing Genomics Institute (Shenzhen, China). Gene model annotation files of Homo sapiens and reference genome sequences were downloaded from NCBI. The raw data were processed via the software SOAPnuke (v1.5.2), HISAT (v2.1.0), and Bowtie2 (v2.2.5). AS QC and QC of alignment of the processed raw data were qualified, RSEM package (v1.2.8) was used to analyze gene expression, and DEGseq package (v1.50.0) was used to identify differentially expressed genes. The raw sequence data reported in this paper have been deposited in the Sequence Read Archive (SRA) in the National Center for Biotechnology Information (Maryland, USA) under the BioProject ID PRJNA941161.

**Proteome and ubiquitinome**

Sample preparation for HPLC-MS/MS

After stable overexpression of USP10 in AGS cells, total protein was extracted with RIPA lysis buffer (C1053, Applygen, Beijing, China) and protease inhibitor cocktail (G2006, Servicebio, Wuhan, China). Protein content was determined with the BCA kit (G2026, Servicebio, Wuhan, China). The extracted protein solution was reduced with 5 mM dithiothreitol for 30 min at 56 °C. Then, the sample was incubated with 11 mM iodoacetamide for 15 min at room temperature in the dark, and diluted to urea concentration less than 2 M. The sample was digested with trypsin (trypsin: protein = 1:50 (w/w)) at 37 °C overnight and then digested with trypsin (trypsin: protein = 1:100 (w/w)) for additional 4 h. The peptides were separated into 60 fractions with a gradient of 8% to 32% acetonitrile (pH 9.0) for 60 min by high pH reverse-phase HPLC fractionation using Agilent 300Extend C18 column (5 μm particles, 4.6 mm ID, 250 mm length, Agilent Technologies Inc., USA). Then, 60 fractions were combined into 4 fractions and dried by vacuum freeze for LC-MS/MS analysis.

HPLC-MS/MS analysis and Mass spectrometry data analysis

The peptides were eluted from a ReproSil-Pur basic C18 column (100 μm ID, 1.9 μm particle size, 250 mm length, Dr. Maisch, Tübingen, Germany) and analyzed using an EASY-nLC 1000 UPLC system followed by a Q Exactive Plus mass spectrometer and Orbitrap Fusion mass spectrometer (Thermo Fisher Scientific, Waltham, MA, USA). The proteome data were acquired by Q Exactive Plus and Oribtrap Fusion with high-resolution MS2 spectrum with a gradient comprised of an increase from 5% to 23% solvent B (90% acetonitrile, 10% H_2_O, 0.1% formic acid) for 40 min, from 23% to 35% in 12 min, and reaching up to 80% in 4 min, then holding at 80% for the last 4 min at a flow rate of 400 nL/min. The ubiquitinome data were acquired by Q Exactive Plus and Oribtrap Fusion with high-resolution MS2 spectrum with a gradient comprised of an increase from 9% to 25% solvent B (90% acetonitrile, 10% H_2_O, 0.1% formic acid) for 40 min, from 25% to 36% in 11 min, and reaching up to 80% in 4 min, then holding at 80% for the last 4 min at a flow rate of 400 nL/min. The electrospray voltage was set at 2.0 kV. Full MS spectra were 350-1800 m/z with a resolution of 70,000. The 10 most intense precursor ions successively entered into the collision cell for the high-energy collision dissociation (HCD) using a normalized collision energy of 28%, and the fragments were detected with a resolution of 17,500 at m/z = 200 or 100. The automatic gain control (AGC) was set at 5×10^4^. Dynamic exclusion duration was 30 s or 15 s.

The MS/MS data were processed using Maxquant software (v1.5.2.8). For database searching, trypsin/P was set as the cleavage enzyme, the missing cleavages were fixed at 2, and all data were searched against the Swissprot Human database (20, 387 sequences). The mass tolerance for precursor ions was 20 ppm in First search and 5 ppm in Main search, and the mass tolerance for fragment ions was 0.02 Da. The modification searching parameters were set as follows: alkylation on cysteine was specified as fixed modification, acetylation and oxidation on methionine, and ubiquitination on lysine were specified as variable modifications. The FDR of proteins and peptide spectra match was adjusted to 1% and the minimum score for modified peptides was set > 40. 1.5-fold-of-change was considered significant for the proteome and ubiquitome data. The model of sequences constituted with amino acids near the ubiquitin sites in all protein sequences was analyzed using MoMo software (V5.0.2).

**Evaluation of immunohistochemical staining**

Immunohistochemical staining was performed according to the standard protocol. Briefly, formalin-fixed and paraffin-embedded sections (4 µm) were deparaffinized and rehydrated, then treated with 3% H_2_O_2_ and subjected to antigen retrieval with Tris/EDTA buffer (pH 9.0). After blocked with 5% bovine serum albumin for 20 min and incubated with DR5 (TNFRSF10B) (diluted 1:1000, ab230969, Abcam, Cambridge, UK) at 4 °C overnight, the sections were incubated with biotinylated-tagged antibodies and peroxidase-labelled streptavidin (UltraSensitiveTM SP [Mouse/Rabbit]) IHC Kit-9710, Maixin Bio, Fuzhou, China) for 15 min at room temperature. Then, the sections were stained with 3, 3′-diaminobenzidine (DAB) and slightly counterstained with hematoxylins. All procedures were the same for the negative controls, but with PBS replacing the primary antibody.

**Animal experiments**

All animal experiments were approved by the Laboratory Animal Ethics Committee of Renmin Hospital of Wuhan University (20230205A) and performed according to the guidelines of animal research. AGS cells were divided into two groups and transfected with si-USP10 or si-NC, respectively, and MKN45 cells were divided into three groups and transfected with si-USP10, si-TNFRSF10B, or si-NC, respectively. Then the transfected cells were cultured at 37 ℃, 5% CO2 condition until the cell confluence reached approximately 90%. Male, 4-week-old BALB/c nude mice were purchased from Shulaibao (Wuhan) Biotechnology Co., Ltd. (Wuhan, China) and housed in the specific pathogen-free environment of the Animal Experimental Center, Renmin Hospital of the Wuhan University. After the cells reached the proper confluence, the mice were randomly divided into two groups or three groups (five mice per group) by random number table method, then the 2×10^6^ transfected cells were injected into nude mice via tail vein (suspended in 100 μL phosphate-buffered saline) of the corresponding group. No blinding was performed through the experiments. Mice’s body weights were determined every two days. After the experimental period, all mice were euthanized under general anesthesia induced by 1% pentobarbital sodium at one high-dose of 50mg · kg^-1^ body weight injected intraperitoneally. Then the lung and liver were collected for H&E staining and microscopic observation. Lung metastasis nodules or liver metastasis nodules were examined. The metastasis nodule diameters were measured using a digital vernier caliper.
